# Diet and trophic ecology of the tiger shark (*Galeocerdo cuvier*) from South African waters

**DOI:** 10.1371/journal.pone.0177897

**Published:** 2017-06-08

**Authors:** Matthew L. Dicken, Nigel E. Hussey, Heather M. Christiansen, Malcolm J. Smale, Nomfundo Nkabi, Geremy Cliff, Sabine P. Wintner

**Affiliations:** 1 KwaZulu-Natal Sharks Board, Umhlanga Rocks, South Africa; 2 Department of Zoology and Entomology, University of Fort Hare, Alice. South Africa; 3 University of Windsor–Biological Sciences, Windsor, Ontario, Canada; 4 Department of Zoology, Nelson Mandela Metropolitan University, Port Elizabeth, South Africa; 5 Port Elizabeth Museum, Humewood, Port Elizabeth, South Africa; 6 Biomedical Resource Unit, University of KwaZulu-Natal, Durban South Africa; Dauphin Island Sea Lab, UNITED STATES

## Abstract

Knowledge of the diet and trophic ecology of apex predators is key for the implementation of effective ecosystem as well as species-based management initiatives. Using a combination of stomach content data and stable isotope analysis (δ^15^N and δ^13^C) the current study provides information on size-based and sex-specific variations in diet, trophic position (TP) and foraging habitat of tiger sharks (*Galeocerdo cuvier*) caught in the KwaZulu-Natal Sharks Board bather protection program. This study presents the longest time-series and most detailed analysis of stomach content data for *G*. *cuvier* worldwide. Prey identified from 628 non-empty stomachs revealed a size-based shift in diet. Reptiles, birds, mysticetes, and large shark species increased in dietary importance with *G*. *cuvier* size, concomitant with a decrease in smaller prey such as batoids and teleosts. Seasonal and decadal shifts in diet driven primarily by changes in the importance of elasmobranchs and mammal (cetacean) prey were recorded for medium sized (150–220 cm) *G*. *cuvier*. Both stomach content and stable isotope analysis indicated that *G*. *cuvier* is a generalist feeder at the population level. Size-based δ^13^C profiles indicated a movement to offshore foraging habitats by larger *G*. *cuvier*. Calculated TP varied by method ranging from 4.0 to 5.0 (TP_SCA_ for stomach contents) and from 3.6 to 4.5 (TP_scaled_ and TP_additive_ for δ^15^N). Large (> 220 cm) *G*. *cuvier* did not feed at discrete trophic levels, but rather throughout the food web. These data provide key information on the ecological role of *G*. *cuvier* to improve the accuracy of regional food web modelling. This will enable a better understanding of the ecological impacts related to changes in the abundance of this predator.

## Introduction

Large sharks are one of the most ecologically important group of animals in coastal and open ocean systems [1, 2, 3]. Overfishing and habitat loss, however, has increasingly resulted in declines in some populations [4, 5, 6]. Sharks can affect the structure and function of the ecosystem at the community level through both direct predation and risk effects [1, 7]. As a result, understanding the potential of top predator removal is critical. Some studies suggest their potential for driving large scale cascading effects within food webs [2, 3, 8] while more recent analysis suggests this may not be the case [9, 10, 11]. To improve our understanding of the wider ecological consequences of predator removal, detailed information is required on species’ ecological roles. In particular, information is required on diet and dietary switches with body size and sex as these ultimately determine the species trophic position [2, 12, 13].

The diet and trophic ecology of large sharks can be assessed using a range of techniques. Each technique, however, has its own limitations and biases, which need to be considered when designing studies and interpreting results. Direct observation of feeding behavior provides the most accurate information on food ingested, however, this is impractical for almost all shark species as they are difficult to observe, highly mobile and wide ranging. Traditionally, stomach content analysis (SCA) has been used to provide an insight into the type and diversity of prey consumed [14, 15, 16]. Although it provides detailed taxonomic resolution of prey, limitations arise due to the snapshot nature of recently consumed prey, regurgitation of prey upon capture, differential digestion rates of prey and the misidentification of prey [12, 17, 18].

More recently, stable carbon and nitrogen isotope analysis (SIA) has emerged as a complementary tool to SCA and has provided new insights into the trophic relationships among sharks and the ecosystems they inhabit [19, 20]. Stable isotope analysis is based on the fact that ratios of carbon (^13^C/^12^C) and nitrogen (^15^N/^14^N) isotopes in a predator’s tissues reflect the isotopic composition of its prey and foraging location over both time and space [19, 21, 22]. Carbon isotopes reflect variation in baseline producers and hence foraging habitat of the predator [23, 24, 25] whereas nitrogen isotopes indicate its relative trophic position (TP) within the food web [13, 26, 27].

Most studies to date utilise the isotopic values within muscle tissue, collected from multiple animals, to examine feeding behaviour at the population level [28, 29]. However, there is growing evidence that individuals within a population may exhibit different dietary preferences and foraging behaviors [21, 29, 30]. Analysis of multiple tissue types with varying turnover rates allows for the investigation of isotopic variation within and among individuals [22, 31, 32]. Determining the proportion of specialist and generalist feeders within a population is required to better understand the full range of trophic roles a population utilises [33, 34].

Tiger sharks (*Galeocerdo cuvier*) are found worldwide in tropical and warm-temperate coastal and pelagic waters [35]. In the West Indian Ocean (WIO), they occur from the Red Sea to the east coast of South Africa, as well as off Madagascar [36]. In South Africa, their principal range extends from the Mozambique border to Cape St Francis [37, 38]. They are one of the largest apex predators growing to at least 550 cm total length (TL) [39, 40, 41] and are known to consume a wide variety of both invertebrate and vertebrate prey [37, 42, 43]. Ontogenetic shifts in their diet have been identified with larger prey becoming more important with increasing shark size [42, 43, 44].

The movement patterns and foraging habitat of *G*. *cuvier* has been studied at various locations including Australia [45, 46], Hawaii [47, 48, 49], the southwest Pacific [50], and the northwest Atlantic [51]. These studies indicate that *G*. *cuvier* utilize large home ranges incorporating a variety of both coastal and oceanic habitats. However, comparatively little is known about the habitat use and trophic ecology of *G*. *cuvier* within the WIO, specifically in South Africa where it is one of the commonly caught species within the bather protection program of the KwaZulu-Natal Sharks Board (KZNSB) [41, 52, 53].

Previous studies utilising SCA to investgate the diet and feeding ecology of *G*. *cuvier* have been limited by either low sample sizes [37, 54], or the inability to identify prey to species level [42, 43, 44]. Studies utilising SIA have investigated the TP of *G*. *cuvier* within the broader context of the large shark assemblage in the WIO [13] and examined size-based variation in inter-tissue isotopic values and individual dietary specialization in Western Australia [22]. Despite this SCA and SIA research, there is still uncertainty related to aspects of size, sex and individual-based variation in the trophic ecology of *G*. *cuvier* in South African waters.

Through access to long term data on tiger shark stomach contents (1983 to 2014) combined with multiple tissues sampled from recent captures (2006 to 2014), this paper provides a detailed investigation of the diet and trophic ecology of *G*. *cuvier* off KwaZulu-Natal (KZN), South Africa. The overall aim of the investigation was to examine size-based and sex-specific variations in diet, TP and foraging habitat of *G*. *cuvier* at the individual and population level through a combined SCA and SIA (δ^15^N and δ^13^C) approach. Given the unique time series of stomach content data, decadal shifts in diet were also investigated. These data will provide knowledge to help future efforts to model the ecosystem consequences of depletions or recoveries of *G*. *cuvier* in the region.

## Materials and methods

### Ethics statement

All research in this investigation was conducted under anually renewed operating (OC/OCS/020) and research permits issued by the Department of Environmental Affairs, South Africa. Samples were collected from dead specimens, caught in the KZN bather protection programme, and hence ethical approval was not required.

### Study site and sample collection

**Fig 1:**
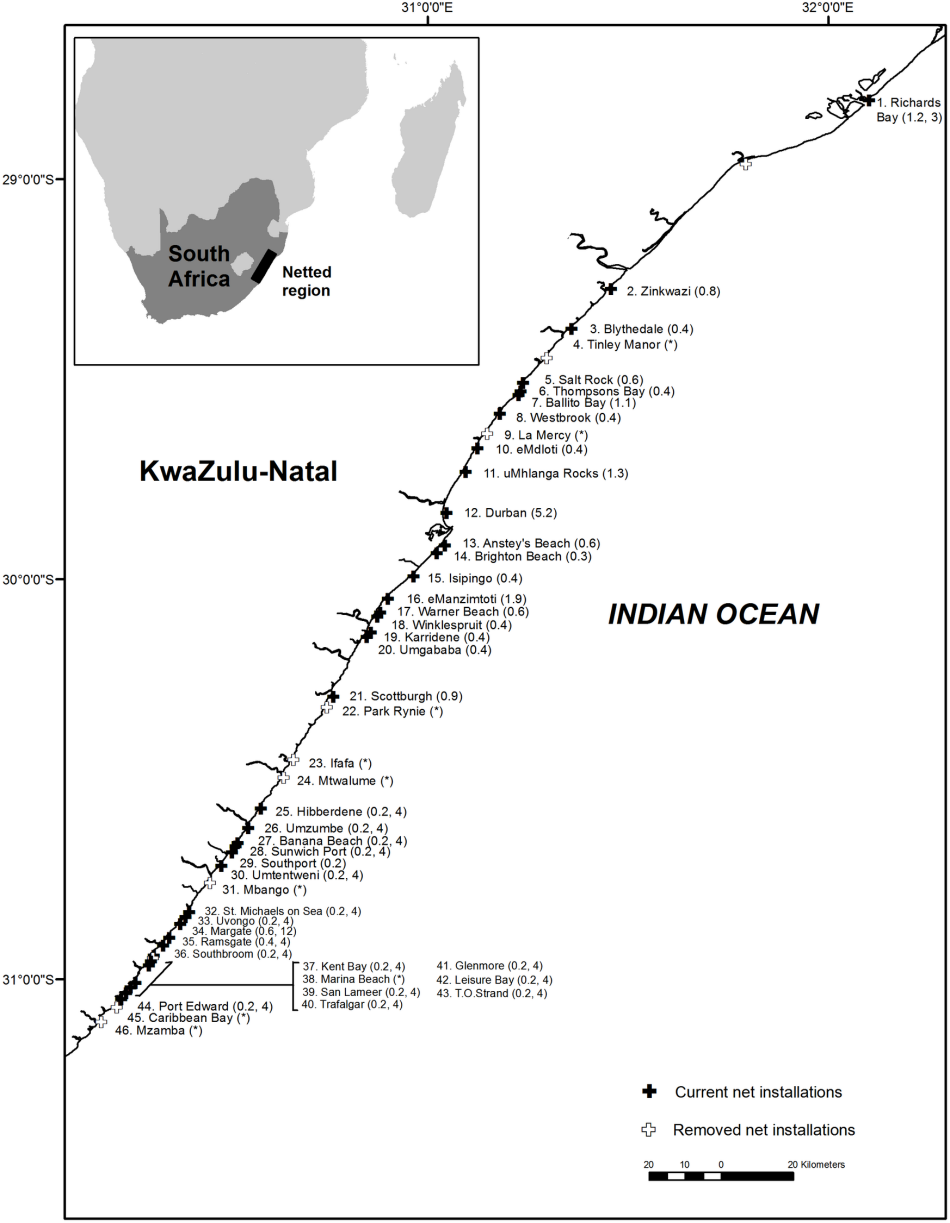
Netted beaches on the KwaZulu-Natal coast and, in parenthesis, the length of nets in kilometres and number of drumlines as of December 2014. Several net installations (*) were removed permanently during the study period 1983–2014. Insert shows the locality of the netted region in relation to the South African coast.

All *G*. *cuvier* were sampled from animals incidentally caught in the KZN bather protection programme. The program currently uses shark nets, or a combination of nets and drumlines at 37 beaches along the KZN coastline (Fig 1). The majority of nets are 213.5 m long, 6.3 m deep, with a stretched mesh of 51cm. All nets are set parallel and approximately 300–500 m from the shore in a water depth of 10–14 m. More details of the netting operation are given by [55]. Drumlines were introduced as a replacement to nets at some installations in 2007. As of December 2014 there were 79 drumlines installed at 18 of the 37 beaches along the coast. Each drumline is anchored adjacent to the nets and consists of a single Mustad 4480DT 14/0 J hook (Gjøvik, Norway) suspended 4 m beneath a large float [56, 57]. The hooks are baited with southern rover (*Emmelichthys nitidus*) or jacopever species (Scorpaenidae).

Recently caught sharks, which were dead but not yet decomposed, were retrieved and transported to the KZNSB laboratory where they were stored frozen (–20°C) until dissection. On arrival at the laboratory, basic data on size, sex, maturity status and morphological measurements, were recorded. Precaudal length (PCL) was measured in centimeters as the straight-line distance between perpendiculars to the snout and the precaudal notch [58]. Maturity status was visually assessed using published criteria [37] and the state of the reproductive organs according to published criteria [59].

### Stomach content analysis

Stomach content data was collected from 1983 to 2014. For specific details on catch rates and seasonality of capture, refer to [41]. For each *G*. *cuvier*, the complete stomach was removed and prey items identified to the lowest possible taxon, counted, and weighed (wet mass) to the nearest 1.0 g. Prey was identified at various levels of digestion including whole animals, teleost otoliths [60] and cephalopod beaks [61, 62]. Cumulative prey curves were constructed to determine if a sufficient number of stomachs had been collected for accurately describing total diet and diet by size class. The order in which the stomachs were analysed was randomised 500 times and the mean cumulative number of new prey items plotted against the number of stomachs sampled.

Diet composition was calculated as percentage number (%N), percentage mass (%M), percentage frequency of occurrence (%F) and percentage index of relative importance (%IRI) of prey, from non-empty stomachs, according to the definitions of [14]. Stomach contents containing prey items in conjunction with shark net twine, which suggested they had been scavenged from the nets, were excluded from all analyses. Otoliths and beaks may remain undigested for long periods of time. As a result, stomachs containing only these hard prey items were considered empty and excluded from the analyses to avoid any bias [63, 64]. This procedure has been followed in all previously published KZNSB dietary studies. The exclusion of cephalopod beaks, however, would result in the loss of a wealth of information on the species of cephalopods consumed by *G*. *cuvier* [64]. As a result, their contribution (%N and %F) to the diet of *G*. *cuvier* were analysed separately.

For statistical analysis, prey items were grouped to family level and then further categorised into eight functional prey groups: elasmobranch, teleost, cephalopod, crustacean, reptile, mammal, bird, and miscellaneous items as defined by [18]. To examine whether *G*. *cuvier* undergo a size-based diet shift, individuals were grouped into three size classes: small (< 150 cm), medium (150–220 cm) and large (> 220 cm). The length frequency distribution of sharks sampled can be found in [42]. These size classes were chosen to enable a comparison with previously published dietary studies on *G*. *cuvier* [42, 43, 44]. Seasonal and decadal shifts in diet, for each size class of shark, were investigated between Summer (December to February), Autumn (March to May), Winter (June to August) and Spring (September to November) and between 1983 to 1992, 1993 to 2003 and 2004 to 2014, respectively.

Dietary index (%F, %M, %N and %IRI) data for each prey group and size class of shark were subjected to nonmetric multidimensional scaling (*n*MDS) ordination. To overcome the problem of low prey diversity in the stomachs sampled, dietary data for groups of animals (approximately 5 sharks per group) were randomly pooled within each size class, herein referred to as dietary samples [65, 66]. Prior to *n*MDS ordination, dietary samples were square root-transformed, and a similarity matrix was constructed using the Bray–Curtis similarity coefficient [67]. A one-way analysis of similarities (ANOSIM) test was then employed to determine any statistical differences in diet composition between size classes [66, 68]. There was no signficant effect of sex on initial ANOSIM tests (%F: *R* = 0.024, *p* = 0.26, %M: *R* = 0.009, *p* = 0.59, and %N: *R* = 0.049, *p* = 0.08); consequently sexes were combined for all analyses. The resultant global R statistic ranges from -1 to +1 and provides a measure of similarity among groups, with 0 indicating no difference in groups. Similarity percentage analysis (SIMPER, [69]) was used to determine the functional prey groups most responsible for any significant multivariate differences in diet between size classes, seasons and decades.

Ordination means plots with approximate 95% regions were constructed through bootstrap averages (n = 100) using metric MDS (*m*MDS) [69]. These plots provide a much clearer and intuitively easier structure to interpret than plots, which contain a point for each dietary sample. Mean plots also have lower stress values (better representation), as well as providing information on the magnitude of differences both between and within group locations. All analyses were performed in PRIMER (PRIMER-E Ltd., Ivybridge, UK).

### Stable isotope analysis

For a subset of *G*. *cuvier* caught between 2006 and 2014 (n = 56), a tissue plug was excised from the white muscle block anterior to the first dorsal fin adjacent to the vertebral column for SIA. For 17 individuals, additional liver and skin samples were excised from the central region of either the right or left liver lobe and anterior of the first dorsal fin, respectively. All samples were immediately stored at -20^°^C. Each sample was then freeze dried and muscle and liver tissue were ground to a fine homologous powder using a hand-held mortar and pestle. For skin tissue, surgical scissors were used to cut the tissue into a fine material. Muscle and liver tissue were lipid extracted (LE) using a modified chloroform methanol treatment outlined by [70], following [20]. Skin tissue was not lipid extracted given expected low lipid content [71]. Following LE, muscle and liver were placed in a fume hood to evaporate remaining chloroform methanol and then freeze dried a second time. While water washing is recomended to remove urea from elasmobranch tissue [70] it was not undertaken in the current study to maintain consistency in sampling protocols with previously archived stable isotope data for this region. In addition, LE is known to remove the majority of urea [71, 72] and a previous SIA examination of sharks caught in the KZNSB nets suggested urea may have started to break down prior to analysis and consequently had limited effect on δ^15^N values (72). Between 400 and 600 μg of each tissue sample was weighed into tin cups and carbon and nitrogen stable isotopes analyzed using a Thermo Finnigan Delta^Plus^ mass-spectrometer (Thermo Finnigan, San Jose, CA, USA) coupled with an elemental analyzer (Costech, Valencia, CA, USA) at the Great Lakes Institute for Environmental Research, Windsor, Canada. Stable isotope results are expressed in standard delta notation (δ; parts per thousand) according to the following equation;

$$\delta =\left[(\frac{{(R}_{sample}}{R_{standard}})-1\right]{\times 10}^{3}$$ (1)

where R is the ratio of heavy to light isotopes in the sample and standards. The standard reference material was atmospheric nitrogen for N_2,_ and Pee Dee Belemnite carbonate for CO_2._ The analytical precision based on the standard deviation of two standards (NIST 8414 and internal fish muscle lab standard; *n* = 76) was 0.10‰ and 0.21‰ for δ^15^N and 0.06‰ and 0.09‰ for δ^13^C, respectively. Analytical accuracy based on the analysis of NIST standards, performed with muscle tissue sample, sucrose (NIST 8542), and bovine liver and muscle samples (*n* = 3 for each), were within 0.07‰ for δ^15^N and 0.01‰ for δ^13^C of certified values.

### Single tissue diet (δ^15^N) and habitat (δ^13^C) ontogenetic profiles

To examine ontogenetic shifts in relative TP (absolute δ^15^N values) and foraging habitat (δ^13^C) of *G*. *cuvier*, muscle isotope data for all individuals (n = 56) were grouped by sex and plotted by size (PCL) and body mass. Both linear and polynomial regression models were then tested to examine significant relationships and the best model fit presented. Both regression types were tested following [22], given the fact that there can be a lag in isotopic tissue incorporation of diet and *G*. *cuvier* have previously been reported to undertake a dietary shift [42, 43, 44]. Mean δ^13^C values for scalloped hammerheads (*Sphyrna lewini)* that have known nursery grounds on the KZN continental shelf were used following [20] as a proxy for the range of expected δ^13^C values for the KZN coastal habitat.

### Multi tissue stable isotope analysis to infer individual and population level feeding behavior

To examine the effects of sex, body size (PCL), body mass, maturity state, tissue type and year of capture on δ^15^N and δ^13^C values, a linear mixed-effects model was constructed for the subset of 17 *G*. *cuvier* with multiple tissue samples. Each isotope was modeled independently with all factors set as fixed effects with the exception of individual shark ID, which was included as a random effect. The optimal model was then identified by conducting sequential likelihood ratio tests. Non-significant fixed effects were removed in a stepwise manner until minimum adequate models containing only significant factors remained. Tissue type included muscle, liver and skin representing different turnover or isotope integration periods. These periods range from more than a year for muscle [29, 73] to approximately 6 months for liver [73]. The current isotope turnover rate of skin is unknown but is thought to lie somewhere between muscle and liver [25, 71]. Given uncertainties over skin turnover rates, two linear models were constructed. The first included only two time points (muscle and liver isotope values per individual) while the second included three time points (muscle, skin and liver per individual). Prior to each model run, all tissue data were standardized to remove the effects of tissue-specific isotope values. LE muscle and liver isotope data were corrected with known diet-tissue discrimination factors (DTDF) of 2.3‰ and 1.5‰, respectively, for δ^15^N, and 0.9‰ and 0.2‰, respectively for δ^13^C, according to [72, 74]. DTDF values for BULK skin isotopes are unknown, but are thought to be similar to cartilage (vertebrae) [20]; therefore DTDFs of 1.5‰ for δ^15^N and 3.8‰ for δ^13^C were used [74].

To further investigate the feeding behavior of *G*. *cuvier* on a generalist to specialist continuum, (i.e. diet consistency of individuals over time), mixed model variance component analysis was used to estimate the total observed variability for the population (total isotopic niche width–TNW) by summing the intercept variability (between individual component–BIC) and residual variability (within individual component—WIC) in the random effect of the linear models above [34, 75]. This was calculated for both sets of models (two and three tissue). BIC indicates the dietary variability among individuals, while WIC indicates dietary consistency of an individual over time (34). Specialist feeding behavior is indicated by a higher BIC than TNW value and generalist behavior vice versa. The absolute measure of individual specialization for all *G*. *cuvier*, measured on a scale of 0–1, was then calculated as the ratio of WIC/TNW. A value of 0 (0%) represents specialized feeding behavior, a value of 1 (100%) generalist feeding behavior and values in between, a continuum between the two end points (i.e., 0–49.9%—specialist and 50–100%—generalist) [34]. Statistical analyses were performed in R v. 3.2.3 (R Development Core Team 2015) using the nlme package v. 3.1–124 [76] with an α of 0.05.

### Trophic position estimation from stomach contents (TP_SCA_) and stable isotope analysis (TP_SIA_)

To estimate TP of *G*. *cuvier* from stomach content analysis (TP_SCA_),—a measure of the position an organism occupies in the food web, the following equation was used [15]:

$${TP}_{SCA}=1+\sum_{i=1}^{7}\;p_{i}\times {TP}_{i}$$ (2)

where TP_SCA_ is diet-calculated TP per dietary sample, *p*_*i*_ is the proportion of each prey category in the total diet (expressed as %M), and *TP*_*i*_ is the TP for each functional prey category. The TP of functional prey categories were defined [15] as birds (3.87), cephalopods (3.20), crustaceans (2.52), elasmobranchs (3.65), mammals (4.02), reptiles (2.40), and teleosts (3.24). The miscellaneous functional prey group was excluded from all TP calculations.

To estimate TP of *G*. *cuvier* using nitrogen stable isotopes, a scaled Δ^15^N framework approach (TP_scaled_) based on a dietary δ^15^N value-dependent model was used [77, 78]. With knowledge of the δ^15^N value of a known baseline consumer (δ^15^N_base_), the δ^15^N value of the consumer (individual *G*. *cuvier*; δ^15^N_consumer_), the dietary δ^15^N value at which δ^15^N incorporation and δ^15^N elimination are equal (δ^15^N_lim_) and the rate at which the ratio between δ^15^N incorporation and δ^15^N elimination changes relative to dietary δ^15^N averaged across the food-web (*k*), TP_scaled_ is calculated as follows:

$${TP}_{scaled}=\frac{\log)\delta^{15}N_{lim}-\delta^{15}N_{base})-log(\delta^{15}N_{lim}-\delta^{15}N_{TP})}{k}+{TP}_{base}$$ (3)

For comparative purposes, *G*. *cuvier* TP was also estimated using a constant discrimination factor of 3.4‰ in an additive framework (TP_additive_) following [79];

$${TP}_{additive}=\left[\frac{{(\delta }^{15}N_{consumer}-\delta^{15}N_{base})}{3.4}\right]+2$$ (4)

Where TP_additive_ is the estimated TP of the consumer of interest, δ^15^N_consumer_ is the δ^15^N value of the individual consumer, δ^15^N_base_ is the δ^15^N value of a baseline species, and 3.4‰ is the fixed discrimination value.

Three baseline species were used to estimate TP_scaled_ and TP_additive_ and an average TP value presented to increase confidence in TP estimation. Baseline species included zooplankton *(*copepod; *Euphausia frigida* and mysid; *Undinula vulgaris;* n = 16; mean δ^15^N ± SD = 5.2‰ +- 0.8), whale sharks *(Rhincodon typus;* n = 3; mean δ^15^N ± SD = 9.9‰ +- 0.5) and manta rays *(*manta sp.: n = 8; mean δ^15^N ± SD = 9.8‰ +- 0.5). Whale sharks and Manta rays, which are known zooplanktivores [80, 81, 82], were included as TP3 baseline consumers (TP = 3), while zooplankton was included as a TP2 consumer (77). For the TP_scaled_ approach, a value of k = 0.14 and δ^15^N_lim_ = 21.9 were used following a meta-analysis of experimental isotopic studies [77, 78].

## Results

### Stomach content analysis

**Fig 2:**
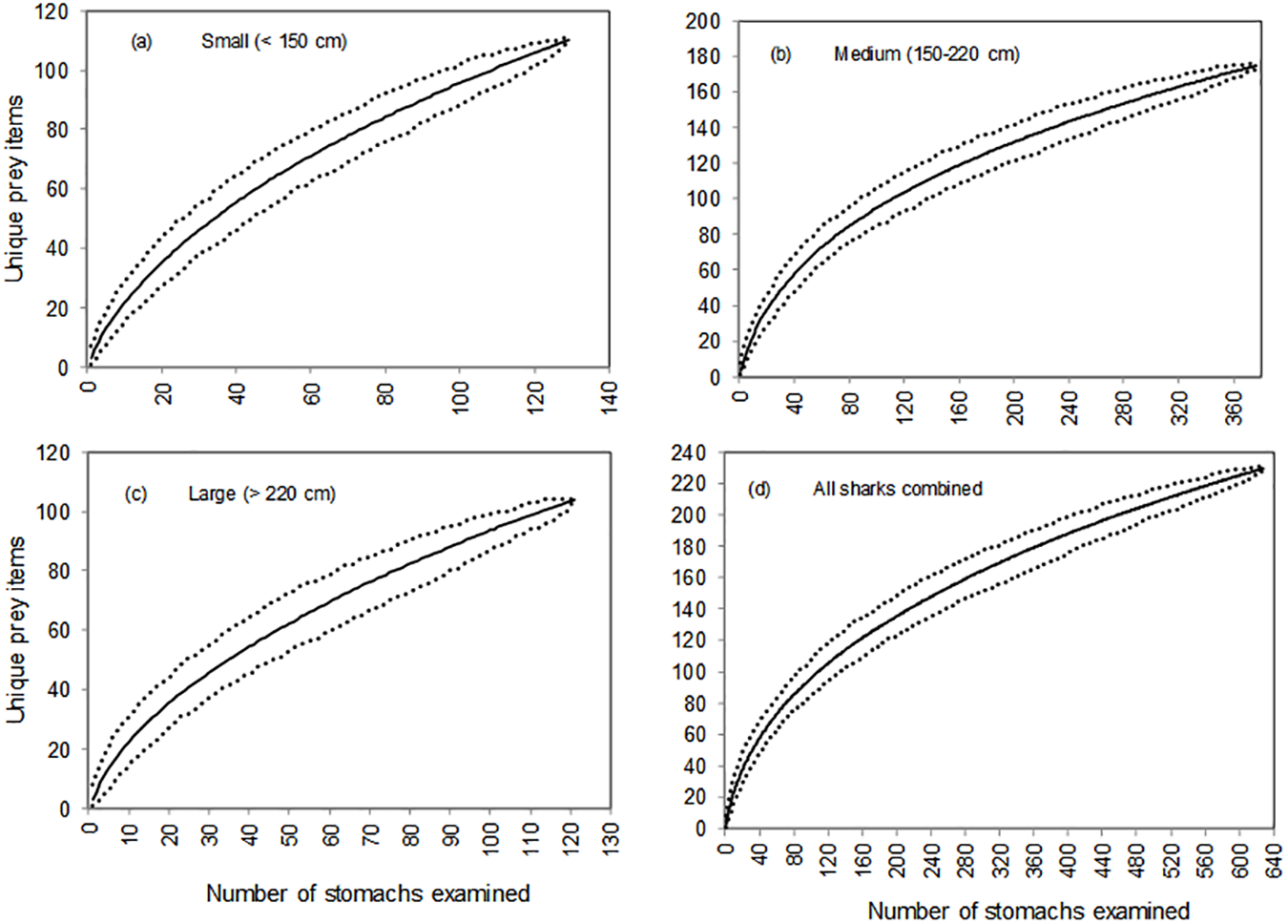
Randomized cumulative prey curves derived from the stomach contents of *G*. *cuvier* caught in the KwaZulu-Natal shark nets and drumlines, 1983–2014. a) Small, b) medium, c) large size classes and d) all sharks combined. The order in which the stomachs were analysed was randomised 500 times and the means (solid lines) and 95% confidence levels (dashed lines) presented.

A total of 778 *G*. *cuvier*, ranging in size from 94 to 335 cm PCL (mean = 185.8 cm, SD = 39.4), were examined. None of these sharks were either neonate, or pregnant and less than 1.5% were considered mature. Of these, 81 (10.4%) had empty stomachs and 69 (8.9%) had regurgitated during capture. Cumulative prey curves were constructed using data from the remaining 628 stomachs that contained food items. When examining each of the size classes as well as all sharks combined, none of the curves (Fig 2A–2D) reached an asymptote indicating that a greater number of individuals would be required to completely describe *G*. *cuvier* diet for this region.

**Fig 3:**
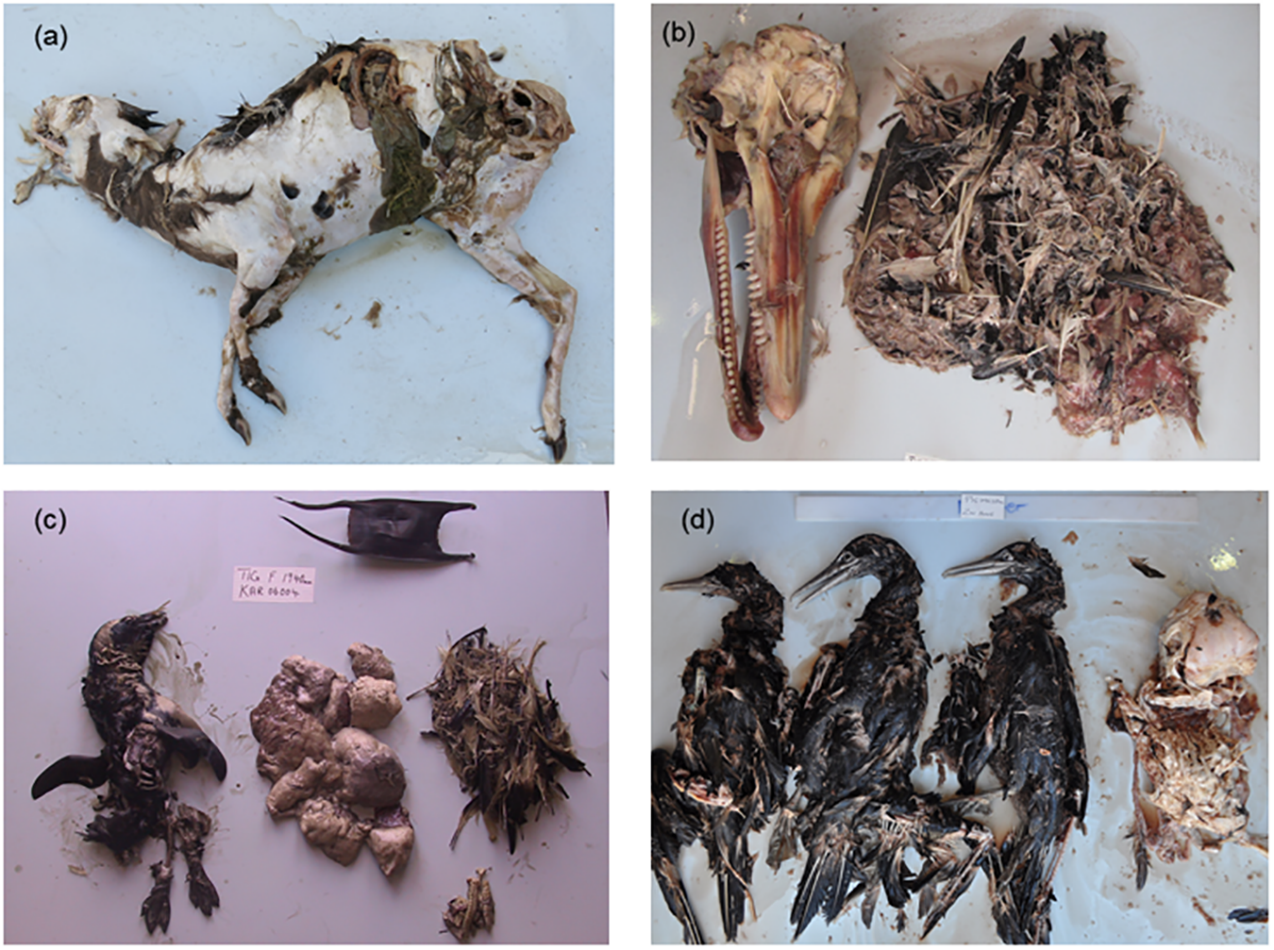
Stomach contents retrieved from *G*. *cuvier* caught in the KwaZulu-Natal shark nets and drumlines, 1983–2014. (a) *Philantomba monticola* (blue duiker) (240 cm female). (b) *Sousa plumbea* (humpback dolphin) and unidentified seabird (195 cm female). (c) *Spheniscus demersus* (African penguin), skate egg case, unidentified shark, *Megaptera novaeangliae* (humpback whale) (194 cm female). (d) *Morus capensis* (Cape gannet) and unidentified porcupine fish (232 cm male).

A diverse range of 192 prey items were identified from the stomach contents of *G*. *cuvier* (S1 Table, Fig 3). Prey items ranged in size from small unidentified shrimps and bivalves to various large whale species including *Physeter macrocephalus* (sperm whale) and *Megaptera novaeangliae* (humpback whale).

Elasmobranchs were the most important functional prey group (%IRI) for medium and large *G*. *cuvier* (Table 1). A total of 20 shark and 18 batoid species were identified (S1 Table). With increasing *G*. *cuvier* body size (small to large), the number of identified shark prey species increased from 5 to 12, whereas the number of batoid prey species decreased from 11 to 7 (S1 Table). Although all size classes of *G*. *cuvier* commonly preyed on unidentified dasyatid (stingray), *Manta birostris* (oceanic manta) and *Carcharhinus obscurus* (dusky shark), the dietary importance of these and other elasmobranch prey varied with size class. There was a general decrease in the importance of typically small inshore elasmobranchs such as dasyatids, *Rhinobatos* sp. (guitarfish), neonate *C*. *obscurus* and an increase in larger and more offshore species such as *Carcharias taurus* (raggedtooth shark), and *Squatina africana* (African angelshark) with increasing *G*. *cuvier* body size (S1 Table).

**Table 1:** Stomach content composition of *G*. *cuvier* caught in the KwaZulu-Natal shark nets and drumlines, 1983–2014. Results are summarized for eight functional prey groups and presented by frequency of occurrence (%F), by mass (%M), by number (N%) and index of relative importance (%IRI). Totals represent number of non-empty stomachs (F), mass prey items (M, kg) and number of unique prey items recorded (N).

				Predator category												
Prey category	All				Small (<150 cm)				Medium (150–220 cm)				Large (>220 cm)			
	*%F*	*%M*	*%N*	*% IRI*	*%F*	*%M*	*%N*	*% IRI*	*%F*	*%M*	*%N*	*% IRI*	*%F*	*%M*	*%N*	*% IRI*
Elasmobranchs	54.74	54.35	14.72	3780.64	44.26	52.79	18.27	3144.97	60.70	55.77	16.07	4361.39	47.11	52.25	9.74	2920.35
Teleosts	51.31	7.44	19.89	1402.25	72.95	18.18	35.60	3923.22	47.15	7.97	14.12	1041.63	42.15	4.73	24.92	1249.52
Reptiles	6.21	1.65	1.60	20.20	1.64	0.21	0.62	1.36	5.69	1.68	1.54	18.32	11.57	1.84	2.08	45.33
Birds	26.96	6.38	6.48	346.64	15.57	3.51	3.72	112.59	27.91	4.40	6.85	313.92	35.54	10.16	7.03	610.97
Mammals	40.69	27.83	9.92	1536.23	29.51	20.99	10.22	920.98	43.36	28.02	10.76	1681.66	44.63	28.70	8.15	1644.40
Cephalopods	15.52	0.86	29.94	478.14	14.75	1.59	12.38	206.22	17.62	1.10	35.43	643.53	10.74	0.33	26.36	286.78
Crustaceans	12.75	0.64	7.86	108.39	9.02	0.50	4.33	43.55	14.36	0.56	7.27	112.44	11.57	0.80	11.02	136.76
Micellaneous items	37.91	0.84	9.59	395.32	54.10	2.23	14.86	924.51	33.88	0.49	7.97	286.41	33.88	1.19	10.70	402.95
Totals	612	1341.59	192		122	80.67	83		369	787.06	148		121	473.85	91	

Teleosts were the most important functional prey group (%IRI) for small *G*. *cuvier* (Table 1). Representatives from 28 families and 67 species were identified (S1 Table). Although a wide variety of prey were consumed many of them had a relatively low incidence. Prey included inshore soft bottom demersal and benthic fishes (e.g. *Galeichthys feliceps*, *Pomadasys olivaceum*), reef associated species (e.g. *Sarpa salpa*, *Epinephelus andersoni*), as well as more offshore epipelagic species (e.g. istiophorid sp., *Thunnus albacares*). The most common species recorded from stomachs (%F and %N) for all size classes of *G*. *cuvier* were from the families Diodontidae (porcupinefish), Tetraodontidae (pufferfish) and Ostraciidae (boxfish). However, the importance of these families in the diet of *G*. *cuvier* decreased with size. Otoliths without any associated soft tissue (excluded from S1 Table) were only identified for single samples of the following: *Otolithes ruber* (snapper kob), *Cheilodonichthys* sp. (gurnards), unidentified synodontid (lizardfish) and unidentified macrourid (grenadiers or rattails). The latter are benthic species typically occurring on the outer-continental shelf and slope at depths of more than 200 m.

Mammals became an increasingly important prey group (%IRI) for both medium and large *G*. *cuvier* (Table 1). A total of 20 prey items was identified representing at least 7 marine and 8 terrestrial species (S1 Table). Small odontocetes (e.g. *Tursiops aduncus* and *Delphinus delphis*) were the most commonly consumed prey in terms of %IRI for small and medium size class *G*. *cuvier*. As body size increased mysticetes (e.g. *Megaptera novaeangliae*) became the more dominant prey. Interesting terrestrial species recorded in stomach contents included: *Cryptomys hottentotus* (Common mole-rat), *Philantomba monticola* (blue duiker) and *Hystrix africaeaustralis* (South African porcupine). Human (*Homo sapiens*) remains, comprising parts of tibia, fibula and pelvis bones were recorded from the stomachs of two sharks with lengths of 2.1 and 2.3 m (S1 Table).

Birds increased in dietary importance (%IRI) with *G*. *cuvier* body size (Table 1) with the Cape gannet (*Morus capensis*) being the most commonly consumed species (S1 Table). Reptiles were one of the least important functional prey groups, however, their importance increased with *G*. *cuvier* body size (Table 1). The most common turtle species were *Chelonia mydas* (green turtle) and *Caretta caretta* (loggerhead turtle). At least two reptile and five bird species recorded had terrestrial origins (S1 Table).

A wide variety of Miscellaneous items were recorded from stomach samples, particularly from small *G*. *cuvier* (S1 Table). Items included unidentified gastropods, molluscs and seaweed as well as junk food (e.g. sweet and potato crisp packets), terrestrial/flood garbage (e.g. condoms, chamois leather, cigarettes) and butcher's bones (e.g. bags of chicken gizzards, cut abattoir bones). Crustaceans, comprising mainly brachyuran crabs, were recorded from all size classes of *G*. *cuvier* in relatively low numbers (Table 1).

Cephalopods were an important functional prey group (%IRI) in all size classes of *G*. *cuvier*, especially medium sized sharks (Table 1). Cuttlefish (Sepiidae) as well as 28 species (14 families) of squid (Teuthoidea) and 5 species (2 families) of octopus (Octopodidae) were identified from beaks (S2 Table). The %F and %N of cuttlefish were similar in all size classes of *G*. *cuvier* whereas the proportion of oceanic squid and neritic octopi species increased and decreased with shark size, respectively (S2 Table). The most commonly recorded squid was *Ancistrocheirus lesueurii* (sharpear enope squid), an oceanic deep water species. Other lower epipelagic to mesopelagic species identified included *Onykia robsoni (*rugose hooked squid), *Sthenoteuthis oulaniensis (*purpleback flying squid) and *Histioteuthis miranda*. The most commonly recorded octopus species was *Octopus cyanea* (big blue octopus), (S2 Table).

### Multivariate analysis of stomach content data

**Fig 4:**
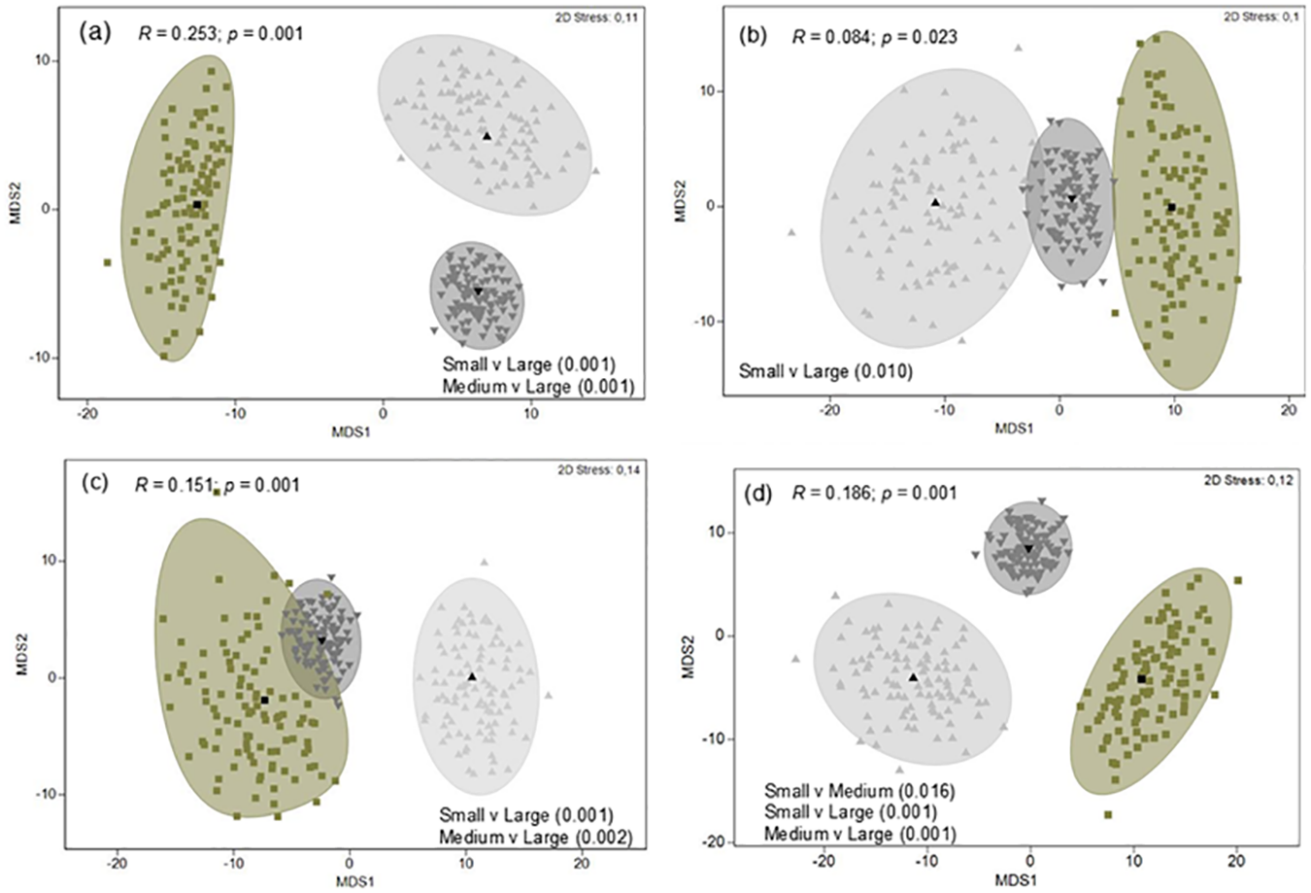
Metric multidimensional scaling (*m*MDS) ordinations of dietary samples with approximate 95% region estimates fitted to bootstrap averages for small (< 150 cm), medium (150–220 cm) and large (> 220 cm) *G*. *cuvier*. (a) Percentage frequency of occurrence (%F), (b) Percentage mass (%M), (c) Percentage number (%N) and (d) Percentage index of relative importance (%IRI). *R*, ANOSIM global *R* statistic and associated *p* value. Significant pairwise tests (with *p* value in brackets) are detailed in each figure.

MDS ordination of dietary samples (small n = 24, medium n = 75 and large n = 24) highlighted a level of dietary separation between small and large *G*. *cuvier*, but the level of separation was moderate, as indicated by the low global R statistic values (Fig 4). ANOSIM pairwise comparisons indicated significant differences between small and large *G*. *cuvier* for all dietary indexes and between medium and large sharks for all indexes except %M. Small and medium sized sharks only exhibited a significant separation for %IRI. Similarity percentage analysis (for all dietary indexes) identified birds, mammals and elasmobranchs as the principal functional prey groups driving this separation.

**Fig 5:**
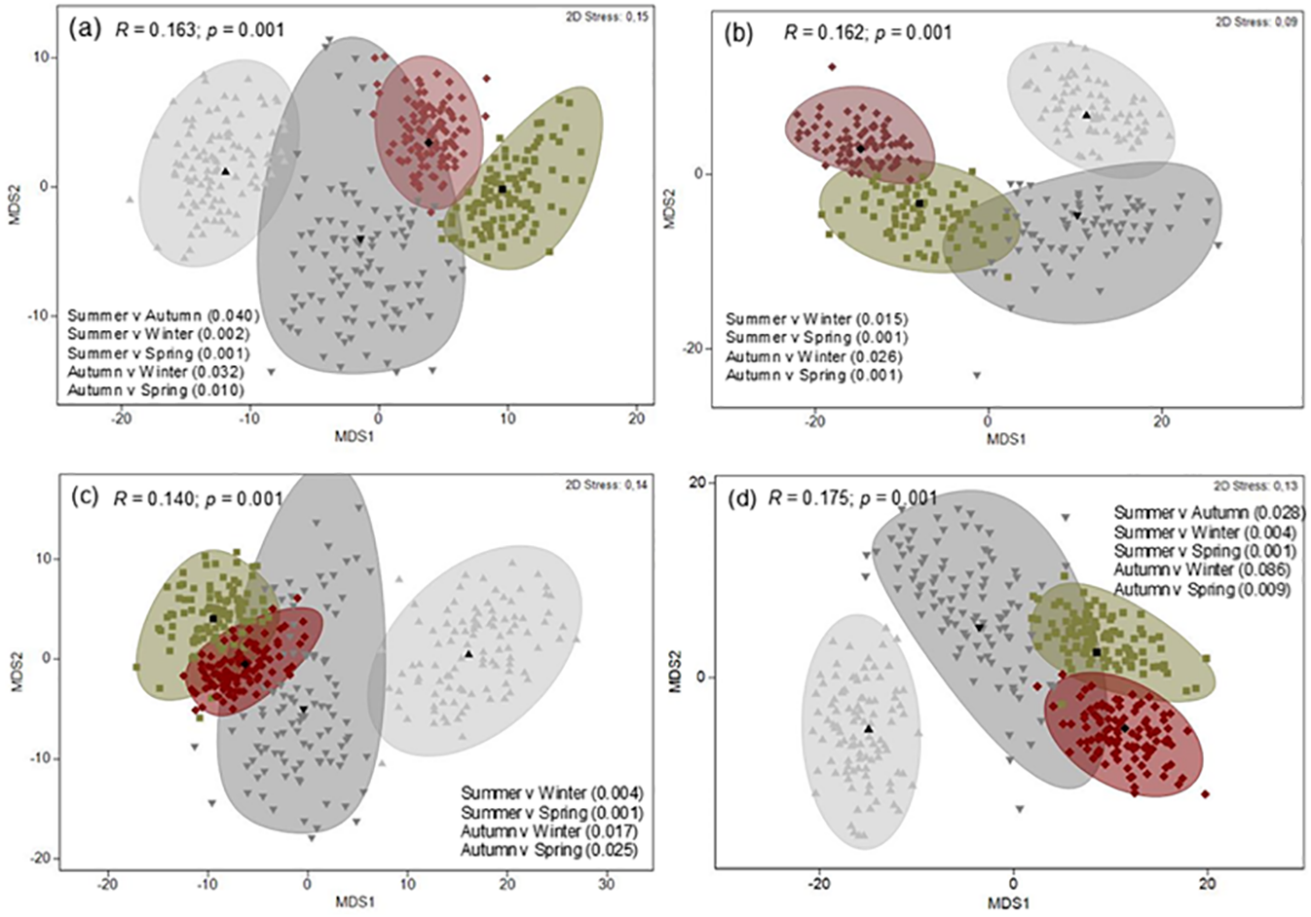
Metric multidimensional scaling (*m*MDS) ordinations of dietary samples with approximate 95% region estimates fitted to bootstrap averages by season for medium (150–220 cm) *G*. *cuvier*. **(**a) Percentage frequency of occurrence (%F), (b) Percentage mass (%M), (c) Percentage number (%N) and (d) Percentage index of relative importance (%IRI). *R*, ANOSIM global *R* statistic and associated *p* value. Significant pairwise tests (with *p* value in brackets) are detailed in each figure.

Seasonal levels of dietary separation were only investigated for medium sized sharks, due to sufficiently large sample sizes (summer n = 21, autumn n = 12, winter n = 14, and spring n = 26). MDS ordination of dietary samples indicated a level of separation (for at least 2 of the dietary indexes) between all seasons except winter and spring (Fig 5). ANOSIM pairwise comparisons indicated these differences were significant. However, a high degree of overlap between all seasons was evident as indicated by the low global *R* statistic values. Similarity percentage analysis (in terms of %IRI) identified elasmobranchs and mammals as the two principal prey groups driving this separation. Elasmobranchs were the dominant dietary component in summer and autumn whereas the importance of mammals increased in winter becoming the dominant group in spring.

**Fig 6:**
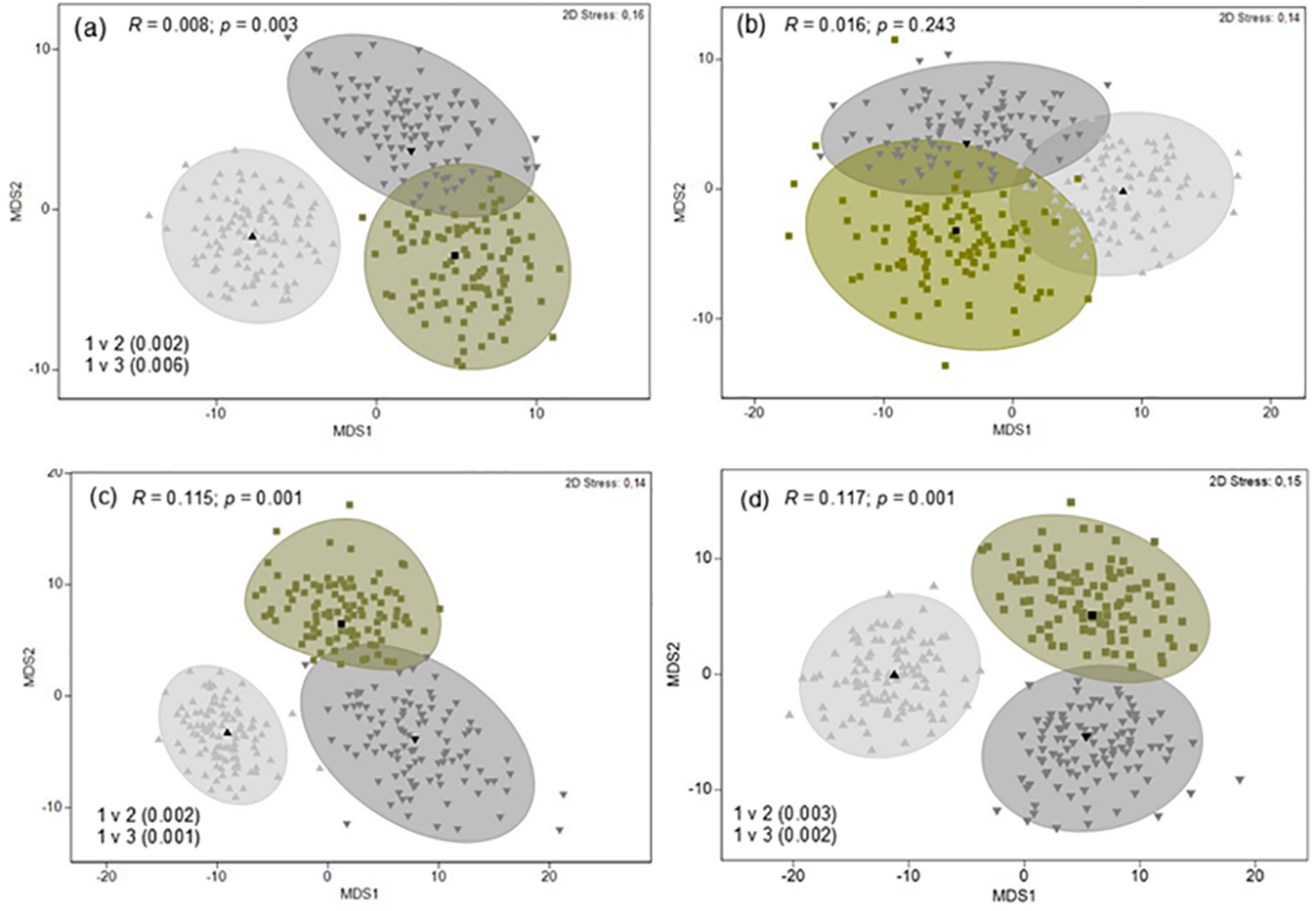
Metric multidimensional scaling (*m*MDS) ordinations of size class 2 (medium) *G*. *cuvier* dietary samples with approximate 95% region estimates fitted to bootstrap averages for decades 1 (1983–1992), 2 (1993–2003) and 3 (2004–2014). (a) Percentage frequency of occurrence (%F), (b) Percentage mass (%M), (c) Percentage number (%N) and d) Percentage index of relative importance (%IRI). *R*, ANOSIM global *R* statistic and associated *p* value. Significant pairwise tests (with *p* value in brackets) are detailed in each figure.

Decadal levels of dietary separation were also only investigated for medium sharks, due to sufficiently large sample sizes (decade 1 n = 30, decade 2 n = 23 and decade 3 n = 21). MDS ordination for all dietary indexes (except %M) indicated a level of separation between decades 1 and 2 and 1 and 3 (Fig 6). ANOSIM pairwise comparisons indicated these differences were significant. However, a high degree of overlap between all decades was evident as indicated by the low global *R* statistic values (Fig 6). Similarity percentage analysis identified elasmobranchs and mammals as the principal functional prey groups driving this separation. Elasmobranchs were the dominant dietary component in Decade 1 whereas the importance of mammals increased in Decades 2 and 3.

### Single tissue diet (δ^15^N) and habitat (δ^13^C) ontogenetic profiles for *G*. *cuvier*

**Fig 7:**
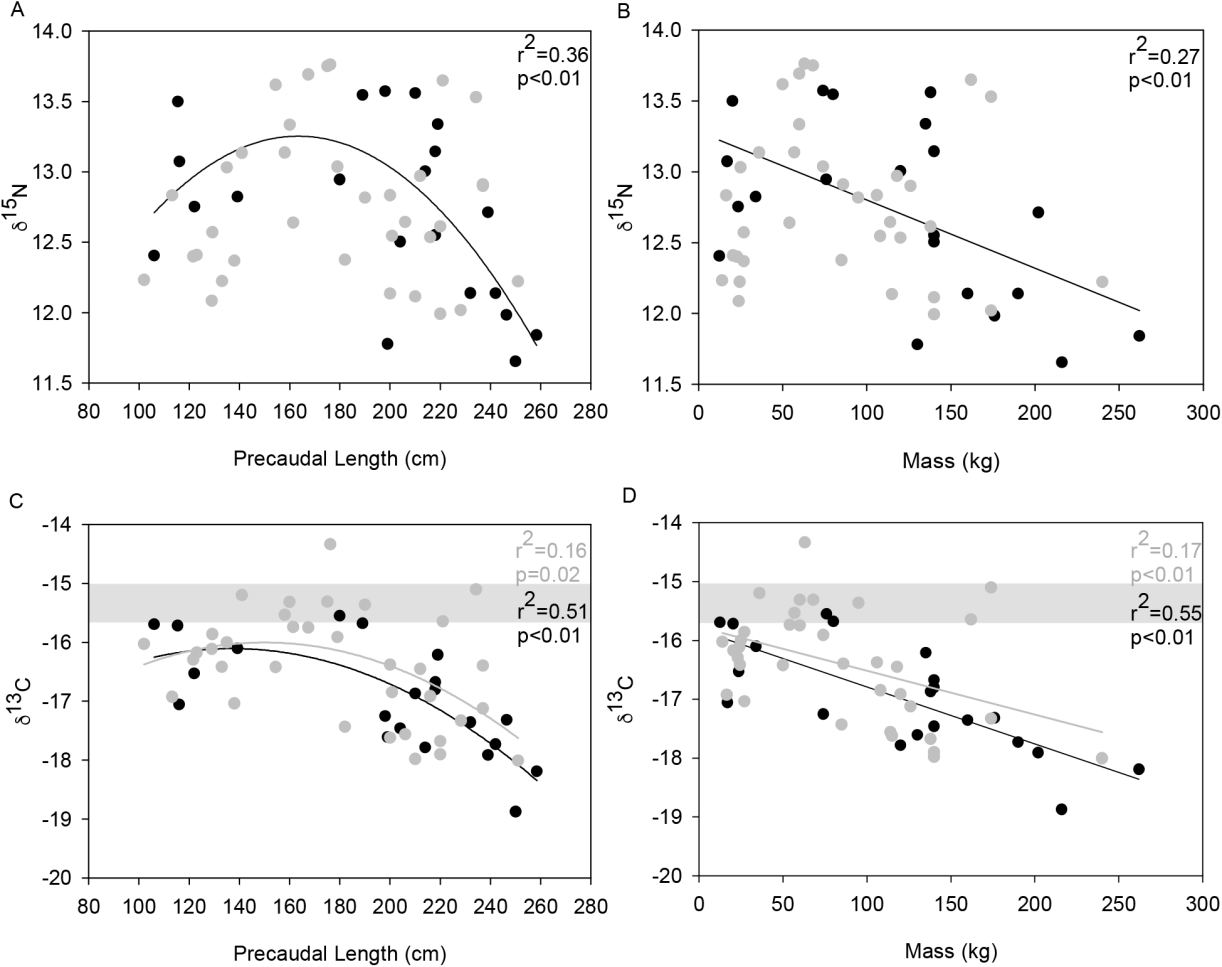
δ^**15**^**N (a and b) and** δ^**13**^**C (c and d) ontogenetic profiles for *G*. *cuvier* by sex (black circles represent males, grey circles represent females).** Linear and polynomial regression models (where appropriate) were fitted to both sexes. Grey bar depicts the predicted δ^13^C range of the KwaZulu-Natal (KZN) coastal habitat of *G*. *cuvier*.

For males, there was a significant relationship between δ^15^N values and PCL (r^2^ = 0.36, p<0.01) that increased until 160–170 cm and then decreased with increasing body size (Fig 7A). There was also a significant negative relationship between δ^15^N and mass (r^2^ = 0.27, p<0.01) for males (Fig 7B). For female sharks, there was no significant relationship between δ^15^N and PCL, or mass. The relationship between δ^13^C and PCL was significant for both sexes, however it was stronger in males (r^2^ = 0.51, *p* < 0.01) than females (r^2^ = 0.16, *p* = 0.02), likely related to sample size (Fig 7C). Similarly, the significant negative relationship between δ^13^C and mass was stronger for males (r^2^ = 0.55, *p*< 0.01) than females (r^2^ = 0.17, *p* < 0.01) (Fig 7D).

### Multi tissue stable isotope analysis to infer individual and population level feeding behavior

Linear mixed-effects models identified that there was a significant effect of tissue type on DTDF corrected *G*. *cuvier* δ^15^N values and a significant effect of PCL on DTDF corrected δ^13^C values for both the two-tissue and three-tissue models (Table 2). All remaining parameters were not significant.

**Table 2:** Results of linear mixed-effects models for *G*. *cuvier* δ^13^C and δ^15^N values for two tissue (muscle and liver) and three tissue (muscle, liver and skin) models with mass, maturity state, precaudal length, sex, tissue and capture year as the fixed effects and shark ID as a random effect. Only significant variables were retained in the optimal model. SE: Standard error.

		Muscle and Liver				Muscle, Liver, and Skin			
		Slope ± SE	df	t-statistic	p-value	Slope ± SE	df	t-statistic	p-value
δ^13^C (‰)									
	Precaudal length	-0.002 ± 0.0002	15	-7.97	<0.01	-0.002 ± 0.0002	15	-7.18	<0.01
δ^15^N (‰)									
	Tissue	0.79 ± 0.11	16	7.19	<0.01	0.80 ± 0.06	33	12.42	<0.01

When δ^13^C and δ^15^N values were both included in the analysis, the total residual variance (WIC) accounted for 60% and 64% of the variation in the two-tissue and three-tissue models, respectively. This indicates that *G*. *cuvier* captured in KZN are generalists (Table 3). When δ^15^N was considered alone the WIC accounted for 42% and 48% of the variation in the two-tissue and three-tissue models, respectively, indicating *G*. *cuvier* are borderline specialists in terms of their diet (Table 3). For δ^13^C, the WIC accounted for 71% and 73% of the variation in the two-tissue and three-tissue models, respectively, indicating *G*. *cuvier* are generalized in terms of foraging location (Table 3).

**Table 3:** Variance component analysis from linear mixed-model analysis for *G*. *cuvier* δ^13^C and δ^15^N for two tissue (muscle and liver) and three tissue (muscle, liver and skin) models. The between-individual component (BIC) represents the total intercept variance and the within-individual component (WIC) represents the residual variance. Total niche width (TNW) is the sum of the intercept and residual variances for δ^13^C and δ^15^N. Total BIC and total WIC are calculated by combining the intercept variances for δ^13^C and δ^15^N and then dividing by TNW. Proportion of WIC and BIC that explained TNW is in parentheses.

	δ^13^C				δ^15^N				Total			
Model	BIC	WIC	TNW	WIC/TNW	BIC	WIC	TNW	WIC/TNW	BIC (%)	WIC (%)	TNW	WIC/TNW
Two Tissue	0.12	0.29	0.41	0.71	0.14	0.10	0.24	0.42	0.26 (40)	0.39 (60)	0.65	0.60
Three Tissue	0.12	0.33	0.45	0.73	0.15	0.14	0.29	0.48	0.27 (36)	0.47(64)	0.74	0.64

### Trophic-level estimation: Stomach contents (TP_SCA_) and δ^15^N (TP_scaled_ and TP_additive_)

**Fig 8:**
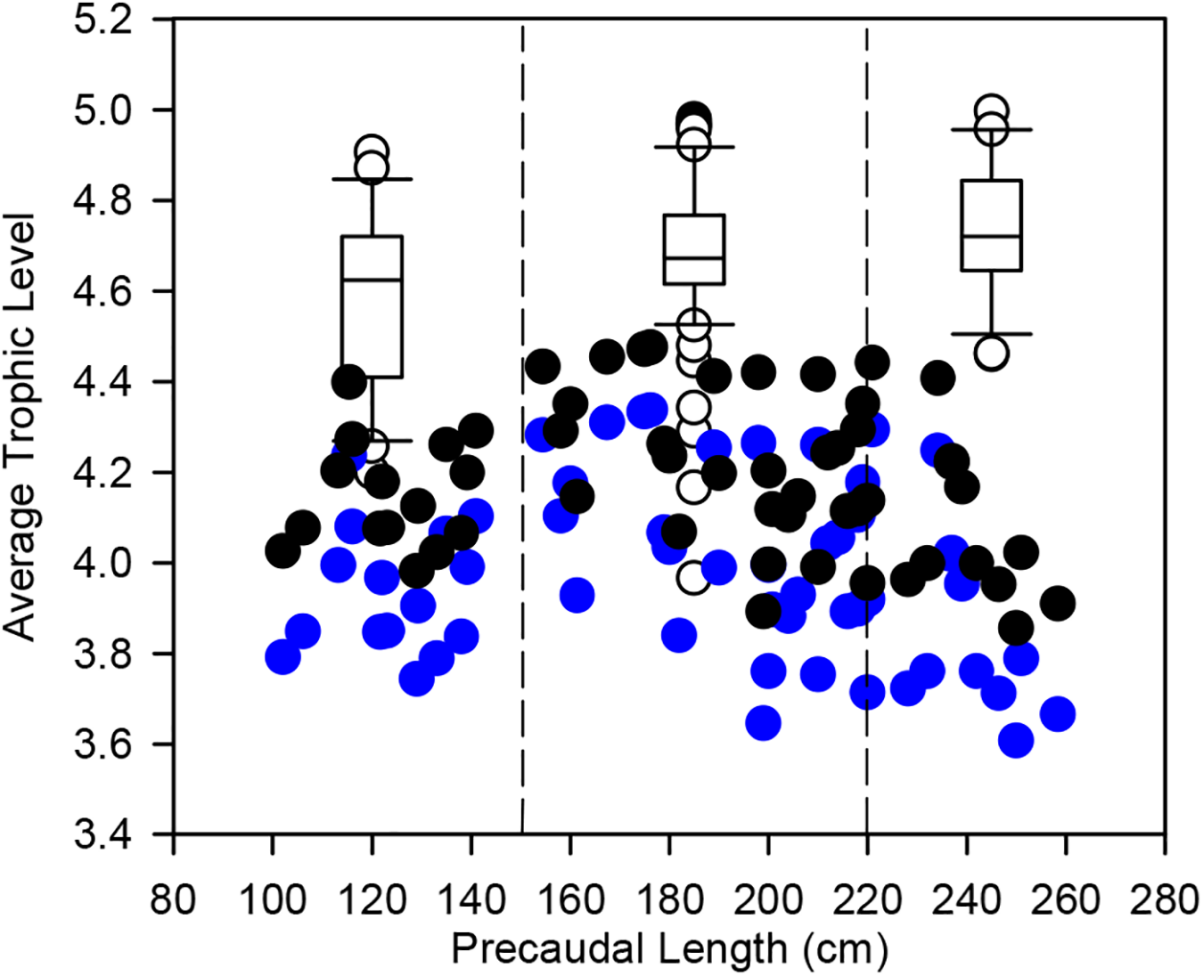
The relationship between TP and increasing body size of *G*. *cuvier*. Stomach content calculated trophic position (TP_SCA_) for each size class is indicated by the white box. The solid black line in each box represents the median, outliers are indicated by open circles. TP estimated using a scaled δ^15^N framework (TP_scaled_) is indicated by blue circles and TP estimated using a standard additive trophic framework (TP_additive_) is indicated by black circles. Vertical dashed black lines indicate the predetermined size classes of *G*. *cuvier* used in the stomach content analysis (<150 cm), medium (150–220 cm) and large (>220 cm).

Overall TP calculated using stomach content data (TP_SCA_) ranged from 4.0 to 5.0 (mean ± SD, 4.7 ± 0.18). There was a minor increase in average TP_SCA_ with increasing body size: small (mean ± SD, 4.6 ± 0.2), medium (mean ± SD, 4.7 ± 0.17), and large (mean ± SD, 4.7 ± 0.14). Overall TP_SCA_ predicted *G*. *cuvier* of all sizes feeding across one trophic level (Fig 8). TP calculated using a scaled Δ^15^N framework (TP_scaled_) predicted that *G*. *cuvier* were feeding across 0.7 of a trophic level with TP ranging from 3.6 to 4.3 (mean ± SD: 4.0 ± 0.2). Estimated TP using the additive framework (TP_additive_) predicted a similar, but slightly higher TP range of 3.9 to 4.5 (range = 0.6 of a TL; 4.3 ± 0.2) (Fig 8).

## Discussion

Stomach content data for *G*. *cuvier* indicates they consume a wide variety of different sized prey of both marine and terrestrial origins along the KZN coast. Although none of the cumulative prey curves in this study reached an asymptote the number of prey items recorded (n = 193) is higher than that recorded for any other species of elasmobranch. From an extensive search of the literature the next highest number of identified prey items (n = 121) was from the dusky shark (*Carcharhinus obscurus*) [58]. In addition, aside from the white shark (*Carcharodon carcharias*) [59, 72, 83], no other shark species has been reported to feed on prey from all eight functional prey groups as defined by [15].

The habitat of the prey, within the functional prey categories, consumed by *G*. *cuvier* was also highly diverse. Teleosts included reef, pelagic and demersal species. Cephalopods and crustaceans included both benthic and pelagic species and reptiles and mammals included terrestrial and marine species. The broad spectrum of prey consumed and the relatively low incidence of most items indicates that *G*. *cuvier* is a generalist feeder, foraging in a variety of different habitats, as previously reported by [43] and [44]. Evidence for this was further provided by the higher intra-individual variation compared to inter-individual variation in δ^13^C values at multiple time scales (i.e. variable tissue turnover rates). This is similar to the generalist strategy previously observed for this species in Australia [30]. Tiger sharks have large home ranges [48, 50, 84] and are known to forage over a wide vertical range exhibiting yo-yo diving behavior [85, 86, 87]. As generalist feeders, these movement patterns likely provide an optimal search strategy to encounter a variety of prey [85], as well as prey with either a low abundance or patchy distribution, which are typical in pelagic waters.

The generalist feeding strategy of *G*. *cuvier*, may in part be related to the seasonal abundance of prey [42, 47, 86]. Interestingly, there was a significant shift in the diet of medium sized *G*. *cuvier* from one dominated by elasmobranchs (in summer and autumn) to one of mammals (mysticetes) (in winter and spring). This coincides with the northward reproductive migration of humpback whales (*Megaptera novaeangliae*) in the winter as they move from the Antarctic to breeding grounds in Mozambique and their return southward migration in the spring [88, 89]. In the North Atlantic and Hawaiian Archipelago *G*. *cuvier* have been shown to switch movement patterns and foraging strategies to take advantage of loggerhead turtles [90] and predictable seasonal congregations of fledging albatross (*Phoebastria* sp.) [48, 91], respectively. In the Abrolhos Islands, Western Australia, *G*. *cuvier* have learnt to exploit the seasonal abundance of discarded bait from the rock lobster fishing industry [44].

Tiger sharks demonstrated asymmetric feeding behavior, whereby larger prey were consumed with increasing predator size, but small prey items were retained in the diet. For example, a variety of both cephalopod and crustacean species were recorded from the stomachs of both small and large *G*. *cuvier* whereas larger prey such as whale sharks and some species of mysticete were found only in large sharks. Although signs of predatory attacks on these larger prey species are rare [92, 93] these events may occur more frequently than existing literature suggests [94, 95, 96]. As such, it is difficult to determine with any certainty whether pieces (up to 14 kg) of these prey items were the result of scavenging or predation. The fact that the diet of small *G*. *cuvier* is a subset of larger individuals is a contributing factor to the high degree of overlap of functional prey categories across size classes. This is similar to that observed for white sharks [72] and most predatory fish [97].

Despite the high degree of dietary overlap between the three size classes there was a clear size based expansion and shift in diet. Reptiles, birds, mysticetes, and large shark species increased in dietary importance with shark size, concomitant with a decrease in smaller prey such as batoids and teleosts. Ontogenetic dietary shifts in *G*. *cuvier*, with larger prey becoming increasingly important with shark size, have been reported in New Caledonia [98], Australia [42, 44] and Hawaii [43]. It has been postulated that these ontogenetic changes are attributable to: 1) larger sharks capable of capturing and consuming larger and more mobile prey [43], 2) increased size of the mouth, jaw and teeth enable the consumption of larger prey and those with a tough shell (e.g. turtles) [44, 99], 3) acquisition of hunting skills required to predate larger, as well as air-breathing prey e.g. turtles and birds [16], and 4) shifts in foraging habitats with shark size [43]. These attributes, together with the fact that human remains were recorded from the stomach contents of a medium sized shark, suggest that *G*. *cuvier* of 150 cm PCL (approximately 203 cm TL) and above are potentially the greatest threat to humans. This is similar to the size of *G*. *cuvier* (230 cm TL) postulated to be the greatest threat in Hawaii [43].

In addition to ontogenetic and seasonal shifts in diet, SCA also indicated a decadal change driven primarily by a decrease in elasmobranch and an increase in mammal (cetacean) prey. Data from the KZNSB bather protection program have shown that there have been declines in several species of sharks, with the exception of *G*. *cuvier*, over the past 30 years, [41, 53]. Declines of shark populations along the coast are likely the result of over-exploitation in a variety of commercial, artisanal and recreational fisheries [100, 101]. Over a similar time period the humpback whale population in the WIO has increased at a rate of 9–11.5% [102]. Although it is difficult to draw strong conclusions from the data available, it is possible, that *G*. *cuvier* (as generalist and opportunistic feeders) are able to take advantage of changes in the relative abundance of different prey through time.

Tiger sharks are regarded as the least discriminate feeders of all shark species [42, 43, 44]. The discovery of a variety of non-digestible anthropogenic as well as digestible terrestrial prey items in this study further confirm its ability to scavenge and forage opportunistically. These attributes may be one of the reasons for the high incidence of ostraciids, tetraodontids and diodontids in their diet. Although of low calorific value these small prey species are likely easy to predate and confer an energetic advantage in achieveing the required daily ration of 0.56% their body weight [103].

It is interesting to note that *G*. *cuvier* is the only species of shark to exhibit a mass capture phenomenon (7 to 14 sharks caught simultaneously at the same netted installation) related to the scavenging of mysticete carcasses in the vicinity of the KZN bather protection nets [41]. It is likely that their foraging strategies confer a competitive advantage over other shark species by benefiting from these unpredictable events and would explain the high dietary occurrence of large prey species such as mysticetes in its diet. Although *G*. *cuvier* are also able to scavenge net-caught manta species the high incidence of shark-inflicted bite marks (76.3%) recorded from reef manta’s (*Manta alfredi*) off Southern Mozambique [104] suggests that most are actively predated.

As *G*. *cuvier* increase in size they range over a wider variety of habitats, which is probably related to the exploration of potential new foraging grounds [41, 49, 86]. Stomach contents indicated that small sharks had a higher proportion of prey typical of inshore and shallow habitats e.g. batoids, benthic octopi and miscellaneous items (transported down rivers) than large sharks. In contrast the stomach contents of larger sharks contained more elasmobranch species, oceanic and deep water squid as well as teleost species typically only found at depths of more than 200 m. These results suggest that larger sharks are spending more time further offshore in the pelagic environment than smaller sharks. This hypothesis is supported by three independent datasets: Firstly, by the fact that very few adult *G*. *cuvier* are caught close inshore in the KZN bather protection programme [41], secondly, by the overall trend of decreasing δ^13^C with increasing size, as offshore waters are typically depleted in δ^13^C [105] and thirdly, tag recaptures of *G*. *cuvier*, indicate they begin to move offshore at a size of about 170 cm PCL [41]. In addition, PCL explained a significant amount of the variability in δ^13^C values providing further support for changes in foraging habitat with size.

Tissue type was the only factor that explained a significant amount of the observed variation in δ^15^N values. Individual tissue types integrate stable isotopes over different time scales, highlighting the requirement to assess diet on long-term scales to fully capture the breadth of a predator’s diet. The slightly higher inter-individual variation observed in δ^15^N than intra-individual variation for both the two-tissue and three-tissue models suggests borderline generalist/specialist feeding behaviour that could be driven by differences between the potential preference of individual *G*. *cuvier* to certain prey types e.g. humpback whales, Cape gannets, shark or batoid species. However, the combined stable isotope results indicate that *G*. *cuvier* along the South African coast is a generalist feeder at the population level, in agreement with stomach content data and previous diet studies [22, 106].

Overall, there was an increasing trend in TP_SCA_ with increasing shark size, as larger *G*. *cuvier* consumed a greater proportion of larger prey from higher trophic levels. This is consistent with ontogenetic shifts recorded in *G*. *cuvier* dietary studies elsewhere in the world [42, 43, 44]. TP_SCA_ calculated from dietary samples, however, varied markedly across the size range of sharks sampled and the size based shift in TP was only slight. This likely reflects the high degree of overlap of functional prey categories consumed across size classes of shark, as well as the wide-ranging movement patterns among various foraging habitats exhibited by *G*. *cuvier*. As a result, changes in the abundance of *G*. *cuvier* may not result in a simple top-down trophic cascade. This highlights the importance of obtaining information on the diet and resource use of *G*. *cuvier*, and indeed any top predator, to better predict the ecological consequences of shark depletions or recoveries [1, 2, 3].

The absolute values of TP_SCA_ estimated for *G*. *cuvier* were similar to those recorded for white sharks in this region [72], supporting the role of this species as a top predator along the South African coastline. Similar to TP_SCA_, both the TP_scaled_ and TP_additive_ estimates increased initially for the small to medium size class *G*. *cuvier*. However, there was then a trend of decreasing TP (for both TP_scaled_ and TP_additive_) from the medium to the large size class of *G*. *cuvier*. The observed decrease in TP_SIA_ for the largest sharks likely represents the long-term incorporation rate of isotope values into muscle tissue and consequently it may represent an integration of variable feeding strategies including foraging in offshore pelagic food webs. Overall, the TP_scaled_ and TP_additive_ estimates were lower and less variable than those of TP_SCA_. SCA represents more recent feeding while SIA integrates dietary data over a longer time frame. Therefore, it is likely that offshore pelagic dietary items (i.e., lower δ^15^N values as a result of lower baseline δ^13^C values in pelagic ecosystems) are better represented by long-term (SIA) data. It is also possible that this results in TP_scaled_ and TP_additive_ underestimating the actual TP value for *G*. *cuvier*, given a single baseline model was used to estimate TP. Alternatively, lower δ^15^N values and associated TP values of larger *G*. *cuvier* could relate to their increased foraging on turtle and certain mammalian prey (for example humpback whales) that typically have lower δ^15^N values. The δ^15^N values for green and loggerhead turtles are approximately 1.7–8.1‰ and 4.0–12.0‰, respectively [107, 108]. The differences observed among these methods to estimate TP highlight the requirement of multiple methods to capture the full breadth of diet and to provide an overall TP range.

**Fig 9:**
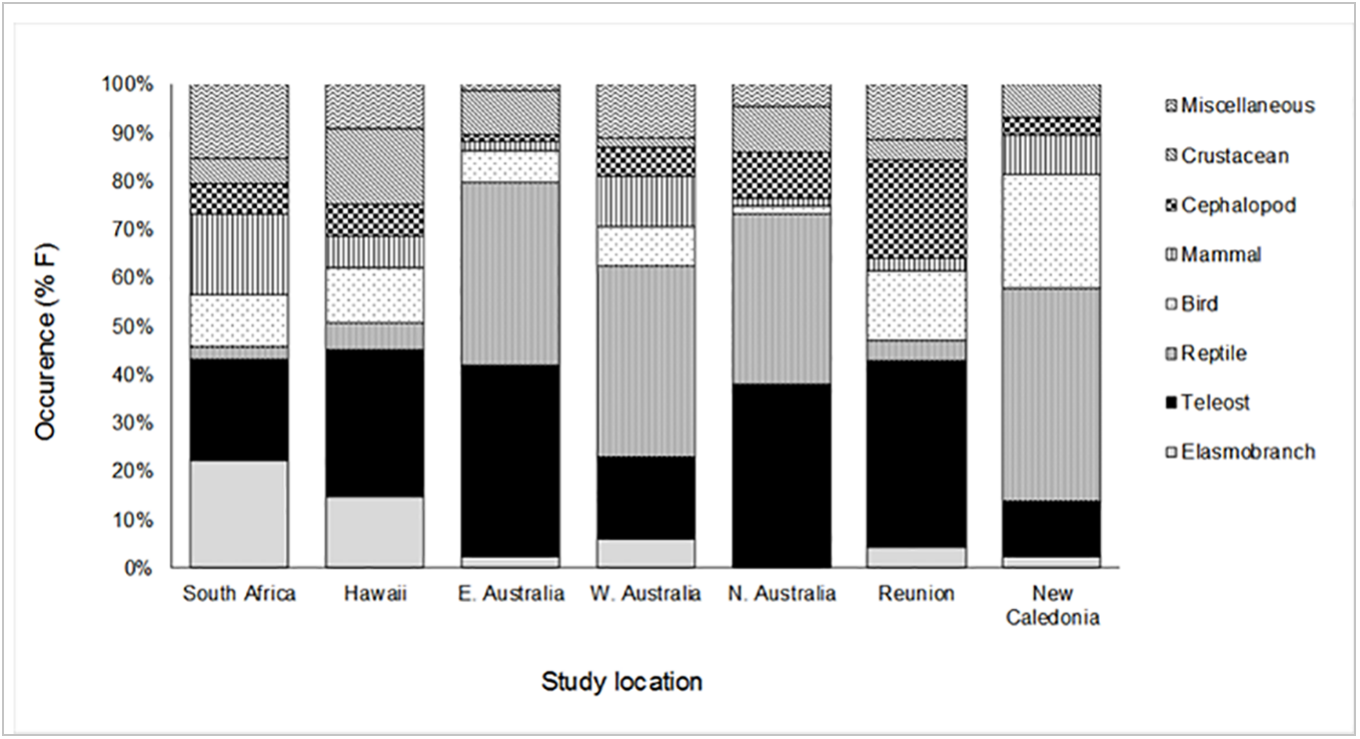
Comparison in the relative percentage frequency of occurrence (%F) of functional prey groups to the diet of *G*. *cuvier* caught in the current study (South Africa) to other geographic regions.

Comparing the findings from diet studies among regions is complicated due to differences in sampling methods e.g. necropsy [42, 43, 44] or regurgitation [54], the habitats they are sampled from e.g. inshore [42, 43] or offshore [44, 109], seagrass meadows [54], or rocky and coral reefs [44, 106] as well as the size and number of sharks sampled. Despite these limitations, distinct differences in the dietary composition of the functional prey groups were evident between regional populations of *G*. *cuvier* (South Africa, Hawaii, Eastern, Western and Northern Australia, Reunion and New Caledonia), (Fig 9).

Although teleosts were an important prey group in all regions, there was a noticeable difference in the importance of elasmobranchs to the diet of *G*. *cuvier* from South Africa and Hawaii, reptiles (turtles and sea snakes) in the Australian and New Caledonia studies and cephalopods from Reunion. In Australia and New Caledonia, *G*. *cuvier* were sampled from regions encompassing large rookeries (and high densities) of both loggerhead and green turtles [110]. In South Africa, Hawaii and Reunion sharks were sampled more than 200 kilometers away from any major turtle nesting sites [110, 111]. In Australia and New Caledonia over 32 and 15 species of sea snakes have been recorded respectively, many of which are known to congregate in shallow coastal waters [112]. In comparison, the only species found in South Africa, Hawaii and Reunion is the yellow-bellied sea snake (*Pelamis platura*), which is pelagic and relatively rare close inshore. As a result, regional differences in diet, at least in part, are a result of differences in the abundance of prey. However, the prevalence of reptiles (especially turtles) in the diet of *G*. *cuvier* from Australia, despite the availability of elasmobranch prey, suggests that diet is not only determined by prey abundance, but also prey preference and catchability. It has been suggested that *G*. *cuvier* are specialist turtle predators [113, 114]. The selective predation of turtles over another abundant species was noted by [44] and postulated to account for the geographic variation in *G*. *cuvier* diet along the west coast of Australia.

This study presents one of the longest time-series and most detailed analysis of stomach content data for *G*. *cuvier* worldwide. It indicates that *G*. *cuvier* in South African waters is a generalist predator, which exhibits an ontogenetic expansion and shift in diet, as previously documented in other geographic localities. There was greater variation in stable isotope values within individual *G*. *cuvier* than among individuals further supporting the concept of a generalist feeding strategy. Stomach content analysis in combination with SIA in this study provides information on the diet and TP for a large apex predator, which exerts influence across multiple components of marine ecosystems. Knowledge of the diet and trophic ecology of *G*. *cuvier* is key for the effective implementation of ecosystem as well as species management initiatives in South Africa and the WIO. The key question that remains, however, is whether the trends observed in this study are indeed indicative of the larger WIO population. This highlights the importance of future studies (such as long-term satellite-tracking) to better understand the level of population connectivity within the region.

## Supporting information

S1 Table Stomach contents of *G*. *cuvier* caught in the KwaZulu-Natal shark nets and drumlines, 1983–2014. (DOCX)

S2 Table Cephalopod species identified from beaks found in the stomach contents of G. cuvier caught in the KwaZulu-Natal shark nets and drumlines, 1983–2014. Details of the prey are presented by frequency of occurrence (%F) and by number (N%). Totals represent number of non-empty stomachs (F) and number of unique prey items recorded (N).
(DOCX)
