# Supplementary material for: Diet and trophic ecology of the tiger shark (Galeocerdo cuvier) from South African waters
Source: PLoS One. 2017 Jun 8;12(6):e0177897. doi: 10.1371/journal.pone.0177897 (PMC5464543; doi:10.1371/journal.pone.0177897)
Supplement: S1 Table — (DOCX) [file pone.0177897.s001.docx]

**S1 Table : Stomach contents of *G. cuvier* caught in the KwaZulu-Natal shark nets and drumlines, 1983–2014.** Details of the prey are presented by frequency of occurrence (%F), by mass (%M), by number (N%) and index of relative importance (%IRI). Totals represent number of non-empty stomachs (F), mass prey items (M, kg) and number of unique prey items recorded (N). Species are shown in phylogenetic order, according to Smith and Heemstra (1986).

|  |  |  |  | **Predator category** | | | | | | | | | | | |  |
| --- | --- | --- | --- | --- | --- | --- | --- | --- | --- | --- | --- | --- | --- | --- | --- | --- |
| **Prey category** | **All** | | |  | **Small (<150 cm)** | | |  | **Medium (150 - 220 cm)** | | |  | **Large (>220 cm)** | | |  |
|  |  |  |  |  |  |  |  |  |  |  |  |  |  |  |  |  |
|  | ***%F*** | ***%M*** | ***%N*** | ***% IRI*** | ***%F*** | ***%M*** | ***%N*** | ***% IRI*** | ***%F*** | ***%M*** | ***%N*** | ***% IRI*** | ***%F*** | ***%M*** | ***%N*** | ***% IRI*** |
|  |  |  |  |  |  |  |  |  |  |  |  |  |  |  |  |  |
| ELASMOBRANCHS | 54.74 | 54.35 | 14.72 | 3780.64 | 44.26 | 52.79 | 18.27 | 3144.97 | 60.70 | 55.77 | 16.07 | 4361.39 | 47.11 | 52.25 | 9.74 | 2920.35 |
| Squalidae |  |  |  |  |  |  |  |  |  |  |  |  |  |  |  |  |
| Unidentified *Squalus* sp. (dogfish) | 0.16 | 0.04 | 0.04 | 0.01 |  |  |  |  | 0.27 | 0.06 | 0.07 | 0.04 |  |  |  |  |
| *Scylliogaleus quecketti* (Flapnose houndshark) | 0.16 | 0.12 | 0.04 | 0.03 |  |  |  |  |  |  |  |  | 0.83 | 0.33 | 0.16 | 0.41 |
| Rhincodontidae |  |  |  |  |  |  |  |  |  |  |  |  |  |  |  |  |
| *Rhincodon typus* (whale shark) | 0.33 | 0.14 | 0.08 | 0.07 |  |  |  |  |  |  |  |  | 1.65 | 0.40 | 0.32 | 1.20 |
| Carcharhinidae (requiem sharks) |  |  |  |  |  |  |  |  |  |  |  |  |  |  |  |  |
| Unidentified carcharhinid | 0.49 | 0.73 | 0.13 | 0.42 |  |  |  |  | 0.54 | 1.02 | 0.14 | 0.63 | 0.83 | 0.37 | 0.16 | 0.43 |
| *C. brevipinna* (spinner shark) | 0.49 | 0.39 | 0.13 | 0.25 | 0.82 | 0.25 | 0.31 | 0.46 | 0.27 | 0.15 | 0.07 | 0.06 | 0.83 | 0.82 | 0.16 | 0.81 |
| *C. obscurus* (dusky shark) | 2.29 | 2.84 | 0.71 | 8.14 | 3.28 | 12.28 | 1.24 | 44.32 | 2.17 | 2.48 | 0.77 | 7.04 | 1.65 | 1.84 | 0.32 | 3.56 |
| *C. sealei (*blackspot shark) | 0.16 | 0.05 | 0.04 | 0.02 |  |  |  |  |  |  |  |  | 0.83 | 0.15 | 0.16 | 0.25 |
| *Galeocerdo cuvier* (tiger shark) | 0.16 | 1.45 | 0.04 | 0.24 |  |  |  |  |  |  |  |  | 0.83 | 4.12 | 0.16 | 3.53 |
| *Loxodon macrorhinus* (sliteye shark) | 0.16 | 0.13 | 0.04 | 0.03 |  |  |  |  | 0.27 | 0.23 | 0.07 | 0.08 |  |  |  |  |
| *Rhizoprionodon acutus* (milkshark) | 0.65 | 0.27 | 0.17 | 0.28 | 0.82 | 0.25 | 0.31 | 0.46 | 0.81 | 0.43 | 0.21 | 0.52 |  |  |  |  |
| Scyliorhinidae |  |  |  |  |  |  |  |  |  |  |  |  |  |  |  |  |
| Unidentified Scyliorhinid (cat shark) | 1.80 | 0.02 | 0.46 | 0.86 | 2.46 | 0.05 | 0.93 | 2.40 | 2.17 | 0.02 | 0.56 | 1.26 |  |  |  |  |
| Sphyrnidae |  |  |  |  |  |  |  |  |  |  |  |  |  |  |  |  |
| Unidentified *Sphyrna* sp. (hammerhead) | 0.16 | 0.85 | 0.04 | 0.15 |  |  |  |  |  |  |  |  | 0.83 | 2.41 | 0.16 | 2.12 |
| *Sphyrna lewini* (scalloped hammerhead) | 0.16 | 0.04 | 0.04 | 0.01 |  |  |  |  | 0.27 | 0.06 | 0.07 | 0.04 |  |  |  |  |
| *S. mokarran* (great hammerhead) | 0.16 | 0.45 | 0.04 | 0.08 |  |  |  |  |  |  |  |  | 0.83 | 1.29 | 0.16 | 1.20 |
| Lamnidae |  |  |  |  |  |  |  |  |  |  |  |  |  |  |  |  |
| *Carcharodon carcharias* (white shark) | 0.16 | 0.12 | 0.04 | 0.03 |  |  |  |  |  |  |  |  | 0.83 | 0.34 | 0.16 | 0.41 |
| *Isurus oxyrinchus* (mako shark) | 0.16 | 0.45 | 0.04 | 0.08 |  |  |  |  | 0.27 | 0.78 | 0.07 | 0.23 |  |  |  |  |
| Odontaspididae |  |  |  |  |  |  |  |  |  |  |  |  |  |  |  |  |
| *Carcharias taurus* (spotted ragged-tooth) | 1.63 | 4.21 | 0.42 | 7.57 | 0.82 | 0.72 | 0.31 | 0.84 | 1.63 | 2.35 | 0.42 | 4.51 | 2.48 | 7.89 | 0.48 | 20.76 |
| Squatinidae |  |  |  |  |  |  |  |  |  |  |  |  |  |  |  |  |
| *Squatina africana* (African angelshark) | 0.65 | 0.56 | 0.17 | 0.47 |  |  |  |  | 0.54 | 0.27 | 0.14 | 0.22 | 1.65 | 1.14 | 0.32 | 2.41 |
| Torpedinidae |  |  |  |  |  |  |  |  |  |  |  |  |  |  |  |  |
| Unidentified *Torpedo* sp. (electric ray) | 0.33 | 0.18 | 0.08 | 0.09 | 0.82 | 0.40 | 0.31 | 0.58 | 0.27 | 0.27 | 0.07 | 0.09 |  |  |  |  |
| *Torpedo sinuspersici* (marbled electric ray) | 0.16 | 0.28 | 0.04 | 0.05 |  |  |  |  | 0.27 | 0.47 | 0.07 | 0.15 |  |  |  |  |
| Rajidae |  |  |  |  |  |  |  |  |  |  |  |  |  |  |  |  |
| Unidentified rajid (skate) | 0.65 | 0.03 | 0.17 | 0.13 | 0.82 | 0.00 | 0.31 | 0.25 | 0.81 | 0.06 | 0.21 | 0.22 |  |  |  |  |
| Rhinobatidae |  |  |  |  |  |  |  |  |  |  |  |  |  |  |  |  |
| Unidentified *Rhinobatos* sp. (guitarfish) | 1.96 | 0.63 | 0.50 | 2.22 | 0.82 | 0.25 | 0.31 | 0.46 | 2.71 | 1.04 | 0.70 | 4.71 | 0.83 | 0.02 | 0.16 | 0.15 |
| *Rhina ancylostoma* (bowmouth guitarfish) | 0.33 | 0.42 | 0.08 | 0.16 |  |  |  |  | 0.54 | 0.72 | 0.14 | 0.46 |  |  |  |  |
| *Rhinobatos annulatus* (lesser guitarfish) | 0.33 | 0.08 | 0.08 | 0.05 |  |  |  |  | 0.54 | 0.13 | 0.14 | 0.15 |  |  |  |  |
| *Rhinobatos leucospilus* (Grayspottted guitarfish) | 0.16 | 0.01 | 0.04 | 0.01 |  |  |  |  | 0.27 | 0.02 | 0.07 | 0.02 |  |  |  |  |
| *Rhynchobatus djiddensis* (giant guitarfish) | 0.98 | 1.84 | 0.21 | 2.01 | 0.82 | 2.70 | 0.31 | 2.47 | 1.08 | 2.59 | 0.21 | 3.04 | 0.83 | 045 | 0.16 | 0.51 |
| Myliobatidae |  |  |  |  |  |  |  |  |  |  |  |  |  |  |  |  |
| Unidentified myliobatid | 0.33 | 0.02 | 0.08 | 0.03 | 0.82 | 0.03 | 0.31 | 0.28 | 0.27 | 0.02 | 0.07 | 0.03 |  |  |  |  |
| *Aetobatus narinari* (spotted eagle ray) | 0.65 | 1.30 | 0.17 | 0.96 |  |  |  |  | 0.81 | 0.50 | 0.21 | 0.58 | 0.83 | 2.85 | 0.16 | 2.49 |
| *Myliobatus aquila* (eagle ray) | 0.16 | 0.08 | 0.04 | 0.02 |  |  |  |  | 0.27 | 0.14 | 0.07 | 0.06 |  |  |  |  |
| *Pteromylaeus bovinus* (bullray) | 0.98 | 1.32 | 0.25 | 1.54 | 0.82 | 6.07 | 0.31 | 5.23 | 1.36 | 1.62 | 0.35 | 2.67 |  |  |  |  |
| *Rhinoptera javanica* (flapnose ray) | 0.33 | 0.84 | 0.08 | 0.30 | 0.82 | 1.09 | 0.31 | 1.15 | 0.27 | 1.32 | 0.07 | 0.38 |  |  |  |  |
| Mobulidae |  |  |  |  |  |  |  |  |  |  |  |  |  |  |  |  |
| *Manta birostris* (oceanic manta) | 4.25 | 12.12 | 1.09 | 56.13 | 1.64 | 7.29 | 0.62 | 12.96 | 5.69 | 15.80 | 1.47 | 98.27 | 2.48 | 6.83 | 0.48 | 18.12 |
| *Mobula eregoodootenkee* (Longhorned devil ray) | 0.82 | 1.77 | 0.21 | 1.62 |  |  |  |  | 0.81 | 1.89 | 0.21 | 1.71 | 1.65 | 1.88 | 0.32 | 3.63 |
| Dasyatidae |  |  |  |  |  |  |  |  |  |  |  |  |  |  |  |  |
| Unidentified dasyatid (stingray) | 7.35 | 4.60 | 2.10 | 49.30 | 8.20 | 4.61 | 3.10 | 63.15 | 8.13 | 5.78 | 2.10 | 64.02 | 4.13 | 2.65 | 1.60 | 17.55 |
| *Dasyatis thetidis* (Black Stingray) | 0.16 | 0.19 | 0.04 | 0.04 |  |  |  |  | 0.27 | 0.32 | 0.07 | 0.11 |  |  |  |  |
| *Himantura draco* (dragon stingray) | 0.16 | 0.17 | 0.04 | 0.03 |  |  |  |  | 0.27 | 0.29 | 0.07 | 0.10 |  |  |  |  |
| *Himantura gerrardi* (sharpnose stingray) | 0.33 | 0.11 | 0.08 | 0.06 | 0.82 | 0.33 | 0.31 | 0.52 |  |  |  |  | 0.83 | 0.24 | 0.16 | 0.33 |
| Gymnuridae |  |  |  |  |  |  |  |  |  |  |  |  |  |  |  |  |
| *Gymnura natalensis* (backwater butterflyray) | 0.49 | 0.72 | 0.13 | 0.41 | 0.82 | 1.79 | 0.31 | 1.72 | 0.54 | 1.04 | 0.14 | 0.64 |  |  |  |  |
| Unidentified shark | 4.74 | 2.36 | 1.30 | 17.35 | 1.64 | 0.79 | 0.62 | 2.31 | 4.88 | 2.44 | 1.40 | 18.71 | 7.44 | 2.49 | 1.44 | 29.22 |
| Unidentified small shark | 5.72 | 0.70 | 1.51 | 12.68 | 4.10 | 0.37 | 1.55 | 7.86 | 5.96 | 0.93 | 1.61 | 15.15 | 6.61 | 0.37 | 1.28 | 10.92 |
| Unidentified large shark | 6.21 | 9.99 | 1.77 | 72.99 | 5.74 | 12.39 | 3.72 | 92.39 | 6.50 | 7.82 | 1.61 | 61.34 | 5.79 | 13.18 | 1.12 | 82.71 |
| Unidentified batoid | 2.12 | 1.18 | 0.59 | 3.77 | 0.82 | 0.79 | 0.31 | 0.90 | 2.98 | 1.91 | 0.84 | 8.19 | 0.83 | 0.05 | 0.16 | 0.17 |
| Unidentified elasmobranch | 4.58 | 0.54 | 1.18 | 7.87 | 4.92 | 0.35 | 1.86 | 10.86 | 5.69 | 0.79 | 1.54 | 13.27 | 0.83 | 0.16 | 0.00 | 0.13 |
| Unidentified elasmobranch egg case | 0.49 | 0.00 | 0.13 | 0.06 | 1.64 | 0.00 | 0.62 | 1.02 | 0.27 | 0.00 | 0.07 | 0.02 |  |  |  |  |
|  |  |  |  |  |  |  |  |  |  |  |  |  |  |  |  |  |
| TELEOSTS | 51.31 | 7.44 | 19.89 | 1402.25 | 72.95 | 18.18 | 35.60 | 3923.22 | 47.15 | 7.97 | 14.12 | 1041.63 | 42.15 | 4.73 | 24.92 | 1249.52 |
| Anguilliformes |  |  |  |  |  |  |  |  |  |  |  |  |  |  |  |  |
| Unidentified anguilliform (eel) | 0.33 | 0.00 | 0.08 | 0.03 |  |  |  |  | 0.27 | 0.00 | 0.07 | 0.02 | 0.83 | 0.00 | 0.16 | 0.13 |
| Congridae |  |  |  |  |  |  |  |  |  |  |  |  |  |  |  |  |
| Unidentified conger eel | 0.16 | 0.00 | 0.04 | 0.01 |  |  |  |  | 0.27 | 0.00 | 0.07 | 0.02 |  |  |  |  |
| Clupeidae |  |  |  |  |  |  |  |  |  |  |  |  |  |  |  |  |
| *Sardinops sagax* (South African sardine) | 0,16 | 0,03 | 4,21 | 0.69 |  |  |  |  |  |  |  |  | 0.83 | 0.08 | 15.97 | 13.27 |
| Ariidae |  |  |  |  |  |  |  |  |  |  |  |  |  |  |  |  |
| Unidentified ariid (sea catfish) | 0.98 | 0.03 | 0.25 | 0.28 | 2.46 | 0.21 | 0.93 | 2.79 | 0.54 | 0.02 | 0.14 | 0.09 | 0.83 | 0.02 | 0.16 | 0.15 |
| *Galeichthys feliceps* (white seacatfish) | 0.49 | 0.09 | 0.13 | 0.10 |  |  |  |  | 0.81 | 0.15 | 0.21 | 0.29 |  |  |  |  |
| Scorpaenidae |  |  |  |  |  |  |  |  |  |  |  |  |  |  |  |  |
| Unidentified *Sebastes* sp.(scorpionfishes) | 0.16 | 0.00 | 0.04 | 0.01 | 0.82 | 0.01 | 0.31 | 0.26 |  |  |  |  |  |  |  |  |
| Platycephalidae |  |  |  |  |  |  |  |  |  |  |  |  |  |  |  |  |
| Unidentified platycephalid (flatheads) | 0.16 | 0.01 | 0.08 | 0.02 | 0.82 | 0.23 | 0.62 | 0.69 |  |  |  |  |  |  |  |  |
| Ambassidae |  |  |  |  |  |  |  |  |  |  |  |  |  |  |  |  |
| Unidentified ambassid (glassfish) | 0.33 | 0.00 | 0.21 | 0.07 | 0.82 | 0.01 | 0.31 | 0.26 | 0.27 | 0.00 | 0.28 | 0.08 |  |  |  |  |
| Serranidae |  |  |  |  |  |  |  |  |  |  |  |  |  |  |  |  |
| Undentified serranid (rockcod) | 0.33 | 0.10 | 0.25 | 0.11 | 1.64 | 1.59 | 1.86 | 5.65 |  |  |  |  |  |  |  |  |
| Unidentified *Acanthistius* sp. (rockcod) | 0.16 | 0.03 | 0.04 | 0.01 |  |  |  |  | 0.27 | 0.05 | 0.07 | 0.03 |  |  |  |  |
| *Epinephelus andersoni* (catface rockcod) | 0.33 | 0.10 | 0.08 | 0.06 |  |  |  |  | 0.54 | 0.17 | 0.14 | 0.17 |  |  |  |  |
| Pomatomidae |  |  |  |  |  |  |  |  |  |  |  |  |  |  |  |  |
| *Pomatomus saltatrix* (elf) | 0.33 | 0.02 | 0.08 | 0.03 |  |  |  |  | 0.54 | 0.03 | 0.14 | 0.09 |  |  |  |  |
| Haemulidae |  |  |  |  |  |  |  |  |  |  |  |  |  |  |  |  |
| Undentified haemulid (rubberlips & grunters) | 0.16 | 0.02 | 0.04 | 0.01 |  |  |  |  | 0.27 | 0.03 | 0.07 | 0.03 |  |  |  |  |
| *Pomadasys commersonnii* (spotted grunter) | 0.65 | 0.83 | 0.38 | 0.79 |  |  |  |  | 1.08 | 1.41 | 0.63 | 2.21 |  |  |  |  |
| *Pomadasys olivaceum* (piggy) | 0.33 | 0.00 | 0.08 | 0.03 |  |  |  |  | 0.27 | 0.00 | 0.07 | 0.02 | 0.83 | 0.00 | 0.16 | 0.14 |
| Sparidae |  |  |  |  |  |  |  |  |  |  |  |  |  |  |  |  |
| Unidentified sparid (seabream) | 0.49 | 0.01 | 0.13 | 0.07 |  |  |  |  | 0.81 | 0.02 | 0.21 | 0.18 |  |  |  |  |
| *Chrysoblephus puniceus* (slinger) | 0.82 | 0.06 | 0.29 | 0.29 | 0.82 | 0.04 | 0.62 | 0.54 | 1.08 | 0.10 | 0.35 | 0.49 |  |  |  |  |
| *Diplodus sargus* (blacktail) | 0.16 | 0.01 | 0.08 | 0.01 | 0.82 | 0.12 | 0.62 | 0.61 |  |  |  |  |  |  |  |  |
| *Pagellus bellottii natalensis* (red tjor-tjor) | 0.33 | 0.01 | 0.17 | 0.06 |  |  |  |  | 0.54 | 0.02 | 028 | 0.16 |  |  |  |  |
| *Sarpa salpa* (strepie) | 0.16 | 000 | 0.04 | 0.01 |  |  |  |  | 0.27 | 0.00 | 007 | 0.02 |  |  |  |  |
| *Rhabdosargus* sp. (stumpnose) | 0.16 | 0.00 | 0.04 | 0.01 |  |  |  |  | 0.27 | 0.00 | 0.07 | 0.02 |  |  |  |  |
| *Acanthopagrus berda* (goldsilk seabream) | 0.16 | 0.00 | 0.04 | 0.01 |  |  |  |  | 0.27 | 0.01 | 0.07 | 0.02 |  |  |  |  |
| *Argyrops filamentosus* (soldier bream) | 0.16 | 0.02 | 0.04 | 0.01 |  |  |  |  | 0.27 | 0.04 | 0.07 | 0.03 |  |  |  |  |
| *Porcostoma dentata* (dane seabream) | 0.16 | 0.02 | 0.04 | 0.01 |  |  |  |  | 0.27 | 0.04 | 0.07 | 0.03 |  |  |  |  |
| *Argyrops spinifer* (king soldier bream) | 0.33 | 0.01 | 0.08 | 0.03 |  |  |  |  | 0.54 | 0.02 | 0.14 | 0.09 |  |  |  |  |
| Ephippidae |  |  |  |  |  |  |  |  |  |  |  |  |  |  |  |  |
| *Platax teira* (longfin batfish) | 0.16 | 0.11 | 0.04 | 0.03 |  |  |  |  | 0.27 | 0.19 | 0.07 | 0.07 |  |  |  |  |
| *Tripterodon orbis* (spade fish) | 0.16 | 0.05 | 0.04 | 0.01 |  |  |  |  | 0.27 | 0.08 | 0.07 | 0.04 |  |  |  |  |
| Sciaenidae |  |  |  |  |  |  |  |  |  |  |  |  |  |  |  |  |
| Unidentified sciaenid (kob) | 0.33 | 0.24 | 0.08 | 0.11 | 0.82 | 3.63 | 0.31 | 3.23 | 0.27 | 0.03 | 0.07 | 0.03 |  |  |  |  |
| *Argyrosomus japonicus* (dusky kob) | 0.49 | 1.23 | 0.13 | 0.66 |  |  |  |  | 0.54 | 1.09 | 0.14 | 0.67 | 0.83 | 1.67 | 0.16 | 1.51 |
| *Argyrosomus thorpei* (squaretail kob) | 0.16 | 0.00 | 0.04 | 0.01 |  |  |  |  | 0.27 | 0.00 | 0.07 | 0.02 |  |  |  |  |
| *Atractoscion aequidens* (geelbek) | 0.49 | 0.05 | 0.13 | 0.09 | 0.82 | 0.18 | 0.31 | 0.40 | 0.54 | 0.06 | 0.14 | 0.11 |  |  |  |  |
| *Johnius amblycephalus* (bellfish) | 0.16 | 0.00 | 0.04 | 0.01 |  |  |  |  | 0.27 | 0.00 | 0.07 | 0.02 |  |  |  |  |
| *Umbrina canariensis* (baardman) | 0.16 | 0.22 | 0.04 | 0.04 |  |  |  |  | 0.27 | 0.38 | 0.07 | 0.12 |  |  |  |  |
| *Umbrina ronchus* (slender baardman) | 0.16 | 0.00 | 0.04 | 0.01 |  |  |  |  | 0.27 | 0.01 | 0.07 | 0.02 |  |  |  |  |
| Oplegnathidae |  |  |  |  |  |  |  |  |  |  |  |  |  |  |  |  |
| Unidentified oplegnathid (knifejaw) | 0.16 | 0.00 | 0.04 | 0.01 | 0.82 | 0.04 | 0.31 | 0.28 |  |  |  |  |  |  |  |  |
| *Oplegnathus conwayi* (Cape knifejaw) | 0,16 | 0,02 | 0,04 | 0.01 |  |  |  |  | 0.27 | 0.03 | 0.07 | 0.03 |  |  |  |  |
| Carangidae |  |  |  |  |  |  |  |  |  |  |  |  |  |  |  |  |
| Unidentified carangid (kingfish) | 0.33 | 0.32 | 0.13 | 0.01 |  |  |  |  | 0.54 | 0.54 | 0.21 | 0.41 |  |  |  |  |
| *Lichia amia* (garrick) | 0.16 | 0.05 | 0.04 | 0.18 |  |  |  |  |  |  |  |  | 0.83 | 0.14 | 0.16 | 0.24 |
| *Trachurus trachurus* (horse mackerel) | 0.16 | 0.01 | 0.08 |  |  |  |  |  | 0.27 | 0.02 | 0.14 | 0.04 |  |  |  |  |
| Coryphaenidae |  |  |  | 0.15 |  |  |  |  |  |  |  |  |  |  |  |  |
| Unidentified coryphaenid (dolphinfish) | 0.16 | 0.12 | 0.04 | 0.01 |  |  |  |  | 0.27 | 0.21 | 0.07 | 0.07 |  |  |  |  |
| Mugilidae |  |  |  | 0.02 |  |  |  |  |  |  |  |  |  |  |  |  |
| Unidentified mugilid (mullet) | 0.16 | 0.04 | 0.04 |  | 0.82 | 0.58 | 0.31 | 0.73 |  |  |  |  |  |  |  |  |
| Gobiidae |  |  |  | 0.03 |  |  |  |  |  |  |  |  |  |  |  |  |
| Unidentified gobiid (gobies) | 0.16 | 0.00 | 0.04 |  |  |  |  |  |  |  |  |  | 0.83 | 0.00 | 0.16 | 0.14 |
| Trichiuridae |  |  |  | 0.01 |  |  |  |  |  |  |  |  |  |  |  |  |
| Unidentified trichiurid (frostfish) | 0.16 | 0.01 | 0.08 |  |  |  |  |  | 0.27 | 0.01 | 0.14 | 0.04 |  |  |  |  |
| *Trichiurus lepturus* (cutlass fish) | 0.16 | 0.02 | 0.04 | 0.01 |  |  |  |  | 0.27 | 0.03 | 0.07 | 0.03 |  |  |  |  |
| Scombridae |  |  |  |  |  |  |  |  |  |  |  |  |  |  |  |  |
| Unidentified scombrid (tuna) | 0.65 | 0.32 | 0.17 | 0.01 | 0.82 | 0.07 | 0.31 | 0.31 | 0.81 | 0.54 | 0.21 | 0.61 |  |  |  |  |
| *Scomber japonicus* (mackerel) | 0.33 | 0.07 | 0.25 | 0.01 |  |  |  |  | 0.54 | 0.13 | 0.42 | 0.30 |  |  |  |  |
| *Scomberomorus plurilineatus* (queen mackerel) | 0.16 | 0.01 | 0.04 |  | 0.82 | 0.09 | 0.31 | 0.33 |  |  |  |  |  |  |  |  |
| *Scomberomorus commerson* (king mackerel) | 0.16 | 0.04 | 0.04 | 0.32 |  |  |  |  | 0.27 | 0.08 | 0.07 | 0.04 |  |  |  |  |
| *Thunnus albacares* (yellowfin tuna) | 0.49 | 0.69 | 0.21 | 0.11 |  |  |  |  | 0.54 | 0.61 | 0.21 | 0.44 | 0.83 | 0.94 | 0.32 | 1.04 |
| Istiophoridae |  |  |  | 0.01 |  |  |  |  |  |  |  |  |  |  |  |  |
| Unidentified istiophorid (billfish) | 0.16 | 0.14 | 0.04 | 0.01 |  |  |  |  |  |  |  |  | 0.83 | 0.41 | 0.16 | 0.47 |
| Pleuronectiformes |  |  |  | 0.44 |  |  |  |  |  |  |  |  |  |  |  |  |
| Unidentified pleuronectiform (flatfish) | 0.16 | 0.00 | 0.04 |  | 0.82 | 0.04 | 0.31 | 0.29 |  |  |  |  |  |  |  |  |
| Soleidae |  |  |  | 0.03 |  |  |  |  |  |  |  |  |  |  |  |  |
| Unidentified soleid (soles) | 0.16 | 0.00 | 0.04 |  |  |  |  |  |  |  |  |  | 0.83 | 0.00 | 0.16 | 0.14 |
| *Austroglossus pectoralis* (East coast sole) | 0.16 | 0.00 | 0.04 | 0.01 | 0.82 | 0.07 | 0.31 | 0.31 |  |  |  |  |  |  |  |  |
| Balistidae |  |  |  |  |  |  |  |  |  |  |  |  |  |  |  |  |
| Unidentified triggerfish | 0.98 | 0.09 | 0.25 | 0.01 | 0.82 | 0.39 | 0.31 | 0.57 | 0.81 | 0.09 | 0.21 | 0.25 | 1.65 | 0.03 | 0.32 | 0.58 |
| *Odonus niger* (redfang trigger fish) | 0.16 | 0.02 | 0.04 | 0.01 |  |  |  |  | 0.27 | 0.03 | 0.07 | 0.03 |  |  |  |  |
| Monacanthidae |  |  |  |  |  |  |  |  |  |  |  |  |  |  |  |  |
| Unidentified monacanthid (filefish) | 0.16 | 0.00 | 0.04 | 0.01 |  |  |  |  | 0.27 | 0.00 | 0.07 | 0.02 |  |  |  |  |
| Thamnaconus arenaceus (sandy filefish) | 0.16 | 0.02 | 0.04 | 0.01 |  |  |  |  | 0.27 | 0.03 | 0.07 | 0.03 |  |  |  |  |
| Ostraciidae |  |  |  |  |  |  |  |  |  |  |  |  |  |  |  |  |
| Unidentified ostraciid (boxfish) | 1.47 | 0.11 | 0.38 | 0.71 | 1.64 | 0.21 | 0.62 | 1.36 | 1.90 | 016 | 0.49 | 1.23 |  |  |  |  |
| *Lactoria fornasini* (thornback cowfish) | 0.16 | 0.02 | 0.04 | 0.01 |  |  |  |  | 0.27 | 0.04 | 0.07 | 0.03 |  |  |  |  |
| *Tetrosomus concatenatus* (triangular boxfish) | 0.49 | 0.05 | 0.13 | 0.09 |  |  |  |  | 0.27 | 0.04 | 0.07 | 0.03 | 1.65 | 0.07 | 0.32 | 0.64 |
| Tetraodontidae |  |  |  |  |  |  |  |  |  |  |  |  |  |  |  |  |
| Unidentified tetraodontid (pufferfish) | 2.94 | 0.10 | 0.80 | 2.63 | 5.74 | 0.33 | 2.17 | 14.36 | 2.44 | 0.12 | 0.70 | 1.99 | 1.65 | 0.02 | 0.32 | 0.57 |
| *Anothron meleagris* (guineafowl pufferfish) | 0.16 | 0.05 | 0.04 | 0.02 |  |  |  |  |  |  |  |  | 0.83 | 0.14 | 0.16 | 0.25 |
| Diodontidae |  |  |  |  |  |  |  |  |  |  |  |  |  |  |  |  |
| Unidentified diodontid (porcupinefish) | 5.56 | 0.12 | 1.43 | 8.59 | 12.30 | 0.85 | 4.64 | 67.49 | 4.34 | 0.10 | 1.12 | 5.27 | 2.48 | 0.02 | 0.48 | 1.25 |
| *Cyclichthys orbicularis* (birdbeak burrfish) | 0.33 | 0.01 | 0.08 | 0.03 | 1.64 | 0.23 | 0.62 | 1.38 |  |  |  |  |  |  |  |  |
| *Diodon hystrix* (spot-fin porcupinefish) | 0.16 | 0.01 | 0.04 | 0.01 |  |  |  |  |  |  |  |  | 0.83 | 0.02 | 0.16 | 0.15 |
| *Lophodiodon calori* (Fourbar porcupinefish) | 0.16 | 0.01 | 0.04 | 0.01 | 0.82 | 0.10 | 0.31 | 0.33 |  |  |  |  |  |  |  |  |
| Unidentified teleost | 23.53 | 1.45 | 7.15 | 199.62 | 33.61 | 5.57 | 16.72 | 715.40 | 19.78 | 1.21 | 5.66 | 135.83 | 24.79 | 1.14 | 5.59 | 166.97 |
|  |  |  |  |  |  |  |  |  |  |  |  |  |  |  |  |  |
| REPTILES | 6.21 | 1.65 | 1.60 | 20.20 | 1.64 | 0.21 | 0.62 | 1.36 | 5.69 | 1.68 | 1.54 | 18.32 | 11.57 | 1.84 | 2.08 | 45.33 |
| Cheloniidae |  |  |  |  |  |  |  |  |  |  |  |  |  |  |  |  |
| Unidentified cheloniid (turtles) | 3.59 | 0.17 | 0.88 | 3.78 | 0.82 | 0.02 | 0.31 | 0.27 | 2.98 | 0.19 | 0.77 | 2.86 | 8.26 | 0.15 | 1.44 | 13.16 |
| *Chelonia mydas* (green turtle) | 1.31 | 0.63 | 0.38 | 1.31 |  |  |  |  | 1.63 | 0.29 | 0.49 | 1.27 | 1.65 | 1.28 | 0.32 | 2.65 |
| *Caretta caretta* (loggerhead turtle) | 0.65 | 0.75 | 0.17 | 0.60 |  |  |  |  | 0.81 | 1.20 | 0.21 | 1.14 | 0.83 | 013 | 0.16 | 0.24 |
| Varanidae |  |  |  |  |  |  |  |  |  |  |  |  |  |  |  |  |
| *Varanus niloticus* (Nile monitor) | 0.49 | 0.11 | 0.13 | 0.12 | 0.82 | 0.19 | 0.31 | 0.41 |  |  |  |  | 0.83 | 0.28 | 0.16 | 0.36 |
| Unidentified land snake | 0.16 | 0.00 | 0.04 | 0.01 |  |  |  |  | 0.27 | 0.00 | 0.07 | 0.02 |  |  |  |  |
|  |  |  |  |  |  |  |  |  |  |  |  |  |  |  |  |  |
| BIRDS | 26.96 | 6.38 | 6.48 | 346.64 | 15.57 | 3.51 | 3.72 | 112.59 | 27.91 | 4.40 | 6.85 | 313.92 | 35.54 | 10.16 | 7.03 | 610.97 |
| Diomedeidae |  |  |  |  |  |  |  |  |  |  |  |  |  |  |  |  |
| *Thalassarche carteri* (Indian yellow-nosed albatross) | 0.16 | 0.18 | 0.04 | 0.04 |  |  |  |  |  |  |  |  | 0.83 | 0.51 | 0.16 | 0.55 |
| Procellariidae |  |  |  |  |  |  |  |  |  |  |  |  |  |  |  |  |
| *Pterodroma macroptera* (great-winged petrol) | 0.16 | 0.04 | 0.04 | 0.01 |  |  |  |  |  |  |  |  | 0.83 | 0.11 | 0.16 | 0.22 |
| Ardeidae |  |  |  |  |  |  |  |  |  |  |  |  |  |  |  |  |
| Undidentifed *ardea* sp. (heron) | 0.16 | 0.09 | 0.04 | 0.02 |  |  |  |  |  |  |  |  | 0.83 | 0.27 | 0.16 | 0.35 |
| Phalacrocoracidae |  |  |  |  |  |  |  |  |  |  |  |  |  |  |  |  |
| Unidentified phalacrocoracid (cormorant) | 0.33 | 0.21 | 0.13 | 0.11 |  |  |  |  | 0.27 | 0.01 | 0.07 | 0.02 | 0.83 | 0.59 | 0.32 | 0.75 |
| Spheniscidae |  |  |  |  |  |  |  |  |  |  |  |  |  |  |  |  |
| *Spheniscus demersus* (African penguin) | 0.49 | 0.21 | 0.13 | 0.16 |  |  |  |  | 0.54 | 0.33 | 0.14 | 0.25 | 0.83 | 0.04 | 0.16 | 0.16 |
| Sulidae |  |  |  |  |  |  |  |  |  |  |  |  |  |  |  |  |
| *Morus capensis* (Cape gannet) | 2.78 | 2.89 | 0.88 | 10.48 | 0.82 | 0.86 | 0.31 | 0.96 | 2.44 | 2.10 | 0.63 | 6.67 | 5.79 | 4.54 | 1.76 | 36.43 |
| Cuculidae |  |  |  |  |  |  |  |  |  |  |  |  |  |  |  |  |
| Unidentifed *Centropus* sp. (coucal) | 0.33 | 0.03 | 0.13 | 0.05 |  |  |  |  | 0.54 | 0.05 | 0.21 | 0.14 |  |  |  |  |
| Columbidae |  |  |  |  |  |  |  |  |  |  |  |  |  |  |  |  |
| Unidentified *Columba* sp. (pigeon) | 0.16 | 0.00 | 0.04 | 0.01 |  |  |  |  | 0.27 | 0.01 | 0.07 | 0.02 |  |  |  |  |
| *Columba livia domestica* (racing pigeon from tags) | 0.33 | 0.04 | 0.08 | 0.04 |  |  |  |  | 0.27 | 0.00 | 0.07 | 0.02 | 0.83 | 0.11 | 0.16 | 0.22 |
| Sturnidae |  |  |  |  |  |  |  |  |  |  |  |  |  |  |  |  |
| *Acridotheres tristis* (Common myna) | 0.16 | 0.01 | 0.04 | 0.01 |  |  |  |  | 0.27 | 0.02 | 0.07 | 0.02 |  |  |  |  |
| Unidentified poultry | 0.33 | 0.09 | 0.08 | 0.06 | 0.82 | 1.30 | 0.31 | 1.32 | 0.27 | 0.03 | 0.07 | 0.03 |  |  |  |  |
| Unidentified bird | 17.97 | 1.84 | 3.91 | 103.37 | 13.93 | 1.35 | 3.10 | 62.00 | 18.70 | 1.27 | 4.40 | 106.10 | 19.83 | 2.87 | 3.19 | 120.24 |
| Unidentified land bird | 1.31 | 0.06 | 0.34 | 0.51 |  |  |  |  | 1.90 | 0.09 | 0.49 | 1.11 | 0.83 | 0.00 | 0.16 | 0.13 |
| Unidentified sea bird | 2.29 | 0.69 | 0.59 | 2.92 |  |  |  |  | 2.44 | 0.49 | 0.63 | 2.73 | 4.13 | 1.13 | 0.80 | 7.98 |
|  |  |  |  |  |  |  |  |  |  |  |  |  |  |  |  |  |
| MAMMALS | 40.69 | 27.83 | 9.92 | 1536.23 | 29.51 | 20.99 | 10.22 | 920.98 | 43.36 | 28.02 | 10.76 | 1681.66 | 44.63 | 28.70 | 8.15 | 1644.40 |
| Unidentified cetacean (dolphins, whales) | 0.33 | 0.02 | 0.04 | 0.02 |  |  |  |  | 0.54 | 0.03 | 0.07 | 0.06 |  |  |  |  |
| Odontoceti |  |  |  |  |  |  |  |  |  |  |  |  |  |  |  |  |
| Unidentified dolphin | 19.28 | 7.14 | 4.79 | 230.04 | 16.39 | 7.43 | 5.88 | 218.28 | 22.76 | 8.62 | 5.66 | 324.99 | 11.57 | 4.63 | 2.24 | 79.44 |
| *Tursiops aduncus* (bottlenose dolphin) | 0.82 | 0.78 | 0.21 | 0.81 | 0.82 | 3.04 | 0.31 | 2.74 | 1.08 | 1.02 | 0.28 | 1.41 |  |  |  |  |
| *Delphinus delphis* (short-beaked common dolphin) | 1.31 | 1.82 | 0.29 | 2.77 | 0.82 | 1.56 | 0.31 | 1.53 | 1.36 | 2.17 | 0.28 | 3.31 | 1.65 | 1.30 | 0.32 | 2.67 |
| *Sousa plumbea* (humpback dolphin) | 0.33 | 0.15 | 0.08 | 0.08 |  |  |  |  | 0.54 | 0.26 | 0.14 | 0.22 |  |  |  |  |
| *Physeter macrocephalus* (sperm whale) | 0.49 | 0.54 | 0.13 | 0.33 |  |  |  |  | 0.54 | 0.19 | 0.14 | 0.18 | 0.83 | 1.22 | 0.16 | 1.14 |
| Mysticeti |  |  |  |  |  |  |  |  |  |  |  |  |  |  |  |  |
| Unidentified whale | 8.01 | 12.59 | 2.06 | 117.32 | 2.46 | 7.64 | 0.93 | 21.08 | 6.78 | 10.99 | 1.75 | 86.28 | 17.36 | 16.10 | 3.35 | 337.64 |
| *Megaptera novaeangliae* (humpback whale) | 0.65 | 1.76 | 0.17 | 1.26 |  |  |  |  | 0.81 | 2.49 | 0.21 | 2.20 | 0.83 | 0.84 | 0.16 | 0.82 |
| *Balaenoptera acutorostrata* (minke whale) | 0.16 | 0.16 | 0.04 | 0.03 |  |  |  |  |  |  |  |  | 0.83 | 0.46 | 0.16 | 0.52 |
| Pinnipedia |  |  |  |  |  |  |  |  |  |  |  |  |  |  |  |  |
| Unidentified pinniped (seals, sealions) | 1.31 | 0.65 | 0.38 | 1.35 | 0.82 | 0.13 | 0.31 | 0.36 | 1.90 | 1.10 | 0.56 | 3.15 |  |  |  |  |
| Bathyergidae |  |  |  |  |  |  |  |  |  |  |  |  |  |  |  |  |
| *Cryptomys hottentotus (*Common mole-rat) | 0.33 | 0.01 | 0.08 | 0.03 |  |  |  |  | 0.27 | 0.01 | 0.07 | 0.02 | 0.83 | 0.02 | 0.16 | 0.15 |
| Suidae |  |  |  |  |  |  |  |  |  |  |  |  |  |  |  |  |
| *Potamochoerus larvatus* (bushpig) | 0.16 | 0.05 | 0.04 | 0.01 | 0.82 | 0.81 | 0.31 | 0.92 |  |  |  |  |  |  |  |  |
| Canidae |  |  |  |  |  |  |  |  |  |  |  |  |  |  |  |  |
| *Canis familiaris* (domestic dog) | 0.16 | 0.01 | 0.04 | 0.01 |  |  |  |  | 0.27 | 0.01 | 0.07 | 0.02 |  |  |  |  |
| Bovidae |  |  |  |  |  |  |  |  |  |  |  |  |  |  |  |  |
| *Capra aegagrus hircus* (domestic goat) | 0.16 | 0.33 | 0.00 | 0.05 |  |  |  |  |  |  |  |  | 0.83 | 0.93 | 0.00 | 0.77 |
| *Philantomba monticola* (blue duiker) | 0.16 | 0.27 | 0.04 | 0.05 |  |  |  |  |  |  |  |  | 0.83 | 0.76 | 0.16 | 0.76 |
| Hystricidae |  |  |  |  |  |  |  |  |  |  |  |  |  |  |  |  |
| *Hystrix africaeaustralis (*porcupine quills*)* | 0.16 | 0.02 | 0.04 | 0.01 |  |  |  |  |  |  |  |  | 0.83 | 0.06 | 0.16 | 0.18 |
| Chiroptera |  |  |  |  |  |  |  |  |  |  |  |  |  |  |  |  |
| Unidentifed bat species | 0.16 | 0.00 | 0.04 | 0.01 | 0.82 | 0.01 | 0.31 | 0.26 |  |  |  |  |  |  |  |  |
| Human remains | 0.33 | 0.12 | 0.04 | 0.05 |  |  |  |  | 0.27 | 0.06 | 0.07 | 0.04 | 0.83 | 0.24 | 0.16 | 0.33 |
| Unidentified land animal | 2.61 | 0.33 | 0.59 | 2.40 | 2.46 | 0.11 | 0.62 | 1.80 | 2.44 | 0.31 | 0.63 | 2.29 | 4.13 | 0.41 | 0.64 | 4.33 |
| Unidentified mammal | 3.76 | 1.07 | 0.80 | 7.03 | 4.10 | 0.25 | 1.24 | 6.12 | 3.79 | 0.76 | 0.84 | 6.06 | 3.31 | 1.73 | 0.48 | 7.31 |
|  |  |  |  |  |  |  |  |  |  |  |  |  |  |  |  |  |
| CEPHALOPODS | 15.52 | 0.86 | 29.94 | 478.14 | 14.75 | 1.59 | 12.38 | 206.22 | 17.62 | 1.10 | 35.43 | 643.53 | 10.74 | 0.33 | 26.36 | 286.78 |
| Sepiidae (cuttlefishes) |  |  |  |  |  |  |  |  |  |  |  |  |  |  |  |  |
| Unidentified sepiid (cuttlefishes) | 10.29 | 0.62 | 27.59 | 290.34 | 7.38 | 0.95 | 8.36 | 68.70 | 1328 | 0.89 | 3403 | 463.72 | 4.96 | 0.11 | 22.68 | 113.04 |
| Teuthoidea |  |  |  |  |  |  |  |  |  |  |  |  |  |  |  |  |
| Unidentified teuthoid (squid) | 0.16 | 0.00 | 0.04 | 0.01 |  |  |  |  | 0.27 | 0.01 | 0.07 | 0.02 |  |  |  |  |
| Ancistrocheiridae |  |  |  |  |  |  |  |  |  |  |  |  |  |  |  |  |
| *Ancistrocheirus lesueurii* (sharpear enope squid) | 0.33 | 0.01 | 0.42 | 0.14 |  |  |  |  | 0.27 | 0.01 | 0.07 | 0.02 | 0.83 | 0.00 | 1.44 | 1.19 |
| Onychoteuthidae |  |  |  |  |  |  |  |  |  |  |  |  |  |  |  |  |
| Onykia *robsoni* (rugose hooked squid) | 0.16 | 0.00 | 0.04 | 0.01 |  |  |  |  | 0.27 | 0.00 | 0.07 | 0.02 |  |  |  |  |
| Chiroteuthidae |  |  |  |  |  |  |  |  |  |  |  |  |  |  |  |  |
| Unidentified *Chiroteuthid* sp. | 0.16 | 0.00 | 0.08 | 0.01 |  |  |  |  | 0.27 | 0.00 | 0.14 | 0.04 |  |  |  |  |
| *Chiroteuthis veranyi* (Verany's long-armed squid) | 0.33 | 0.00 | 0.13 | 0.04 |  |  |  |  | 0.27 | 0.00 | 0.07 | 0.02 | 0.83 | 0.00 | 0.32 | 0.26 |
| Octopodidae |  |  |  |  |  |  |  |  |  |  |  |  |  |  |  |  |
| Unidentified *Octopus* sp. (octopus) | 2.29 | 0.08 | 1.18 | 2.88 | 5.74 | 0.53 | 3.41 | 22.58 | 1.08 | 0.04 | 0.49 | 0.58 | 2.48 | 0.07 | 1.60 | 4.13 |
| *Octopus cyanea* (big blue octopus) | 0.33 | 0.06 | 0.08 | 0.05 |  |  |  |  | 0.27 | 0.01 | 0.07 | 0.02 | 0.83 | 0.15 | 0.16 | 0.25 |
| *Octopus cf vulgaris* (common octopus) | 0.49 | 0.05 | 0.13 | 0.09 |  |  |  |  | 0.54 | 0.09 | 0.14 | 0.12 | 0.83 | 0.01 | 0.16 | 0.14 |
| *Velodona togata* | 0.16 | 0.02 | 0.04 | 0.01 |  |  |  |  | 0.27 | 0.03 | 0.07 | 0.03 |  |  |  |  |
| Unidentified cephalopod | 0.82 | 0.02 | 0.21 | 0.19 | 1.64 | 0.11 | 0.62 | 1.20 | 0.81 | 0.02 | 0.21 | 0.18 |  |  |  |  |
|  |  |  |  |  |  |  |  |  |  |  |  |  |  |  |  |  |
| CRUSTACEANS | 12.75 | 0.64 | 7.86 | 108.39 | 9.02 | 0.50 | 4.33 | 43.55 | 14.36 | 0.56 | 7.27 | 112.44 | 11.57 | 0.80 | 11.02 | 136.76 |
| Brachyura |  |  |  |  |  |  |  |  |  |  |  |  |  |  |  |  |
| Unidentified brachyuran (crabs) | 7.52 | 0.46 | 5.51 | 44.84 | 3.28 | 0.07 | 1.86 | 6.31 | 8.94 | 0.45 | 4.47 | 44.00 | 7.44 | 0.54 | 9.74 | 76.49 |
| Unidentified *Ocypode* sp. (ghost crab) | 0.16 | 0.00 | 0.08 | 0.01 |  |  |  |  |  |  |  |  | 0.83 | 0.01 | 0.32 | 0.27 |
| Unidentified *charybdis* sp. (swimming crab) | 0.16 | 0.01 | 0.13 | 0.02 |  |  |  |  |  |  |  |  | 0.83 | 0.04 | 0.48 | 0.43 |
| *Scylla serrata* (mud crab) | 0.33 | 0.06 | 0.08 | 0.05 |  |  |  |  | 0.27 | 0.01 | 0.07 | 0.02 | 0.83 | 0.15 | 0.16 | 0.26 |
| *Ovalipes punctatus (*sand crab) | 0.16 | 0.00 | 0.04 | 0.01 |  |  |  |  | 0.27 | 0.00 | 0.07 | 0.02 |  |  |  |  |
| Macrura |  |  |  |  |  |  |  |  |  |  |  |  |  |  |  |  |
| Unidentified crayfish | 1.63 | 0.07 | 0.38 | 0.73 | 1.64 | 0.37 | 0.62 | 1.62 | 1.90 | 0.06 | 0.42 | 0.90 | 0.83 | 0.03 | 0.16 | 0.16 |
| *Scyllarides elizabethae* (Shoveller Crayfish) | 0.16 | 0.00 | 0.04 | 0.01 |  |  |  |  | 0.27 | 0.01 | 0.07 | 0.02 |  |  |  |  |
| *Panulirus homarus* (East coast rock lobster) | 0.16 | 0.00 | 0.04 | 0.01 | 0.82 | 0.05 | 0.31 | 0.29 |  |  |  |  |  |  |  |  |
| Caridea |  |  |  |  |  |  |  |  |  |  |  |  |  |  |  |  |
| Unidentified prawn | 0.49 | 0.00 | 0.13 | 0.06 | 0.82 | 0.00 | 0.31 | 0.25 | 0.54 | 0.01 | 0.14 | 0.08 |  |  |  |  |
| Stomatopoda |  |  |  |  |  |  |  |  |  |  |  |  |  |  |  |  |
| Unidentified mantis shrimp | 0.65 | 0.01 | 1.14 | 0.75 | 0.82 | 0.01 | 0.93 | 0.77 | 0.81 | 0.02 | 1.68 | 1.38 |  |  |  |  |
| Unidentified crustacean | 1.31 | 0.02 | 0.29 | 0.41 | 1.64 | 0.00 | 0.31 | 0.51 | 1.36 | 0.01 | 0.35 | 0.49 | 0.83 | 0.03 | 0.16 | 0.16 |
|  |  |  |  |  |  |  |  |  |  |  |  |  |  |  |  |  |
| MISCELLANEOUS ITEMS | 37.91 | 0.84 | 9.59 | 395.32 | 54.10 | 2.23 | 14.86 | 924.51 | 33.88 | 0.49 | 7.97 | 286.41 | 33.88 | 1.19 | 10.70 | 402.95 |
| Unidentified bivalve | 0.16 | 0.00 | 0.04 | 0.01 | 0.82 | 0.00 | 0.31 | 0.25 |  |  |  |  |  |  |  |  |
| Unidentified gastropod | 1.63 | 0.02 | 0.46 | 0.79 | 4.92 | 0.32 | 2.17 | 12.23 | 0.54 | 0.00 | 0.14 | 0.08 | 1.65 | 0.00 | 0.32 | 0.53 |
| Undientifed *Oliva* sp. (olive shells) | 0.16 | 0.00 | 0.04 | 0.01 |  |  |  |  | 0.27 | 0.00 | 0.07 | 0.02 |  |  |  |  |
| Unidentified hydroid | 0.16 | 0.00 | 0.00 | 0.00 | 0.82 | 0.02 | 0.00 | 0.02 |  |  |  |  |  |  |  |  |
| Unidentified mollusc | 0.33 | 0.00 | 0.04 | 0.01 | 1.64 | 0.00 | 0.31 | 0.51 |  |  |  |  |  |  |  |  |
| Unidentified porifera/coelent/ascidia/bryozoa | 0.82 | 0.00 | 0.00 | 0.00 | 2.46 | 0.00 | 0.00 | 0.00 | 0.54 | 0.00 | 0.00 | 0.00 |  |  |  |  |
| Unidentified invertebrate | 0.82 | 0.00 | 0.08 | 0.07 | 1.64 | 0.00 | 0.31 | 0.51 | 0.54 | 0.00 | 0.00 | 0.00 | 0.83 | 0.00 | 0.16 | 0.13 |
| Unidentified plankton | 0.16 | 0.05 | 0.04 | 0.01 |  |  |  |  | 0.27 | 0.08 | 0.07 | 0.04 |  |  |  |  |
| Unidentified seaweed | 6.86 | 0.04 | 1.43 | 10.09 | 11.48 | 0.65 | 3.41 | 46.59 | 5.15 | 0.00 | 1.12 | 5.77 | 7.44 | 0.00 | 1.12 | 8.32 |
| Butcher's bones | 5.56 | 0.62 | 2.35 | 16.54 | 3.28 | 0.56 | 0.93 | 4.87 | 5.96 | 0.30 | 1.40 | 10.09 | 6.61 | 1.18 | 5.27 | 42.65 |
| Junk food | 2.61 | 0.07 | 0.55 | 1.61 | 3.28 | 0.17 | 0.62 | 2.59 | 2.98 | 0.09 | 0.70 | 2.36 | 0.83 | 0.01 | 0.16 | 0.14 |
| Tins | 0.16 | 0.00 | 0.04 | 0.01 |  |  |  |  |  |  |  |  | 0.83 | 0.00 | 0.16 | 0.13 |
| Plastic | 6.54 | 0.01 | 2.23 | 14.61 | 4.10 | 0.00 | 2.17 | 8.88 | 7.59 | 0.01 | 2.73 | 20.76 | 5.79 | 0.00 | 1.12 | 6.47 |
| Terrestrial/flood garbage | 4.74 | 0.00 | 1.43 | 6.79 | 7.38 | 0.00 | 3.41 | 25.12 | 4.07 | 0.01 | 0.98 | 4.00 | 4.13 | 0.00 | 1.44 | 5.94 |
| Unidentified anthropogenic item | 0.49 | 0.03 | 0.08 | 0.06 | 1.64 | 0.50 | 0.62 | 1.84 | 0.27 | 0.00 | 0.00 | 0.00 |  |  |  |  |
| KZNSB twine | 1.96 | 0.00 | 0.17 | 0.33 | 1.64 | 0.00 | 0.00 | 0.00 | 2.17 | 0.00 | 0.28 | 0.61 | 1.65 | 0.00 | 0.00 | 0.00 |
| Sand | 2.45 | 0.00 | 0.04 | 0.10 | 6.56 | 0.00 | 0.00 | 0.00 | 1.90 | 0.00 | 0.07 | 0.13 |  |  |  |  |
| Stone | 1.96 | 0.00 | 0.55 | 1.07 | 2.46 | 0.00 | 0.62 | 1.52 | 1.36 | 0.00 | 0.35 | 0.47 | 3.31 | 0.00 | 0.96 | 3.17 |
| Fisherman's bait | 0.33 | 0.00 | 0.00 | 0.00 |  |  |  |  | 0.27 | 0.00 | 0.07 | 0.02 | 0.83 | 0.00 | 0.00 | 0.00 |
|  |  |  |  |  |  |  |  |  |  |  |  |  |  |  |  |  |
| Totals | 612 | 1341.59 | 192 |  | 122 | 80.67 | 83 |  | 369 | 787.06 | 148 |  | 121 | 473.85 | 91 |  |
