# Supplementary material for: Diet and trophic ecology of the tiger shark (Galeocerdo cuvier) from South African waters
Source: PLoS One. 2017 Jun 8;12(6):e0177897. doi: 10.1371/journal.pone.0177897 (PMC5464543; doi:10.1371/journal.pone.0177897)
Supplement: S2 Table — Details of the prey are presented by frequency of occurrence (%F) and by number (N%). Totals represent number of non-empty stomachs (F) and number of unique prey items recorded (N). (DOCX) [file pone.0177897.s002.docx]

**S2 Table: Cephalopod species identified from beaks found in the stomach contents of G. cuvier caught in the KwaZulu-Natal shark nets and drumlines, 1983–2014. Details of the prey are presented by frequency of occurrence (%F) and by number (N%). Totals represent number of non-empty stomachs (F) and number of unique prey items recorded (N).**

|  | **Predator category** | | | | | | | |
| --- | --- | --- | --- | --- | --- | --- | --- | --- |
| **Prey category** | **All** | | **Small (<150 cm)** | | **Medium (150 - 220 cm)** | | **Large (>220 cm)** | |
|  |  |  |  |  |  |  |  |  |
|  | ***%F*** | ***%N*** | ***%F*** | ***%N*** | ***%F*** | ***%N*** | ***%F*** | ***%N*** |
|  |  |  |  |  |  |  |  |  |
| SEPIOIDEA (cuttlefishes) |  |  |  |  |  |  |  |  |
| Unidentified sepiid | 0.58 | 47.22 | 0.65 | 48.68 | 0.54 | 43.90 | 0.60 | 45.11 |
|  |  |  |  |  |  |  |  |  |
| TEUTHOIDEA (squid) |  |  |  |  |  |  |  |  |
| Unidentified teuthoid | 0.20 | 12.74 | 0.10 | 3.07 | 0.20 | 14.60 | 0.36 | 19.02 |
| Loliginidae |  |  |  |  |  |  |  |  |
| Unidentified Loliginidae (loliginid squids) | 0.05 | 0.95 | 0.08 | 2.63 | 0.03 | 0.60 | 0.04 | 1.09 |
| *Uroteuthis duvaucellii* (indian squid) | 0.02 | 0.41 | 0.03 | 0.88 | 0.02 | 0.30 | 0.02 | 0.54 |
| Enoploteuthidae |  |  |  |  |  |  |  |  |
| Unidentified A*bralia* sp. | 0.01 | 0.14 |  |  | 0.01 | 0.20 |  |  |
| Ancistrocheiridae |  |  |  |  |  |  |  |  |
| *Ancistrocheirus lesueurii* (sharpear enope squid) | 0.23 | 9.28 | 0.08 | 2.63 | 0.27 | 10.90 | 0.27 | 11.96 |
| Octopoteuthidae |  |  |  |  |  |  |  |  |
| Unidentifed O*ctopoteuthis* sp. | 0.09 | 3.73 |  |  | 0.12 | 4.90 | 0.11 | 3.26 |
| *Octopoteuthis sicula* (Ruppell's octopus squid) | 0.02 | 0.54 | 0.05 | 1.32 | 0.02 | 0.40 | 0.02 | 0.54 |
| *Taningia danae* (Dana octopus squid) | 0.00 | 0.07 |  |  | 0.01 | 0.10 |  |  |
| Onychoteuthidae |  |  |  |  |  |  |  |  |
| Unidentified O*nychoteuthis* sp | 0.00 | 0.07 |  |  | 0.01 | 0.10 |  |  |
| Onykia *robsoni* (rugose hooked squid) | 0.03 | 0.95 | 0.02 | 0.88 | 0.03 | 0.90 | 0.04 | 1.63 |
| Pholidoteuthidae |  |  |  |  |  |  |  |  |
| *Pholidoteuthis massyae* | 0.01 | 0.14 | 0.02 | 0.44 | 0.01 | 0.10 |  |  |
| Lycoteuthidae |  |  |  |  |  |  |  |  |
| *Lycoteuthis lorigera* | 0.00 | 0.07 |  |  |  |  | 0.02 | 0.54 |
| Histioteuthidae |  |  |  |  |  |  |  |  |
| Unidentified *Histioteuthis* sp. | 0.02 | 0.47 |  |  | 0.03 | 0.70 |  |  |
| *Histioteuthis hoylei* (flowervase jewel squid) | 0.01 | 0.34 |  |  | 0.02 | 0.50 |  |  |
| *Histioteuthis cf. corona* | 0.01 | 0.75 |  |  | 0.01 | 0.90 | 0.02 | 1.09 |
| *Histioteuthis macrohista* (jewel squid) | 0.01 | 0.34 | 0.03 | 1.32 | 0.01 | 0.20 |  |  |
| *Histioteuthis miranda* | 0.06 | 1.42 | 0.02 | 0.44 | 0.07 | 1.80 | 0.04 | 1.09 |
| Architeuthidae |  |  |  |  |  |  |  |  |
| *Architeuthis dux (giant squid)* | 0.00 | 0.68 |  |  | 0.01 | 1.00 |  |  |
| Ommastrephidae |  |  |  |  |  |  |  |  |
| Unidentified O*mmastrephidae* | 0.01 | 0.14 | 0.02 | 0.44 |  |  | 0.02 | 0.54 |
| Unientified T*odarodes* sp. | 0.00 | 0.07 | 0.02 | 0.44 |  |  |  |  |
| *Ornithoteuthis volatilis (shiny bird squid)* | 0.00 | 0.07 | 0.02 | 0.44 |  |  |  |  |
| *Sthenoteuthis oulaniensis* (purpleback flying squid) | 0.01 | 0.14 | 0.03 | 0.88 |  |  |  |  |
| Chiroteuthidae |  |  |  |  |  |  |  |  |
| Unidentified C*hiroteuthidae*. | 0.02 | 0.61 | 0.02 | 0.44 | 0.03 | 0.80 |  |  |
| *Chiroteuthis veranyi* (Verany's long-armed squid) | 0.07 | 2.51 | 0.03 | 0.88 | 0.08 | 3.10 | 0.07 | 2.17 |
| Mastigoteuthidae |  |  |  |  |  |  |  |  |
| Unidentified M*astigoteuthis* sp. (whip-lash squid) | 0.00 | 0.14 |  |  | 0.01 | 0.20 |  |  |
| Cycloteuthidae |  |  |  |  |  |  |  |  |
| *Discoteuthis discus (rounded disc-fin squid)* | 0.00 | 0.07 |  |  | 0.01 | 0.10 |  |  |
| Cranchidae |  |  |  |  |  |  |  |  |
| Unidentified C*ranchidae* | 0.00 | 0.07 |  |  |  |  | 0.02 | 0.54 |
| Unidentified M*egalocranchia* sp. | 0.00 | 0.07 |  |  | 0.01 | 0.10 |  |  |
| Unidentified T*euthowenia* sp. | 0.00 | 0.07 |  |  | 0.01 | 0.10 |  |  |
|  |  |  |  |  |  |  |  |  |
| OCTOPODA |  |  |  |  |  |  |  |  |
| Octopodidae |  |  |  |  |  |  |  |  |
| Unidentified *Octopus* sp. (octopus) | 0.36 | 13.62 | 0.47 | 31.58 | 0.37 | 11.60 | 0.22 | 7.07 |
| *Octopus cyanea* (big blue octopus) | 0.04 | 1.08 | 0.05 | 2.19 | 0.03 | 0.90 | 0.04 | 1.09 |
| *Octopus cf vulgaris* (common octopus) | 0.03 | 0.68 | 0.02 | 0.44 | 0.03 | 0.80 | 0.02 | 0.54 |
| *Velodona togata* | 0.00 | 0.07 |  |  | 0.01 | 0.10 |  |  |
| Argonautidae |  |  |  |  |  |  |  |  |
| *Argonauta argo* (greater argonaut) | 0.00 | 0.07 |  |  | 0.01 | 0.10 |  |  |
|  |  |  |  |  |  |  |  |  |
| Unidentified cephalopod | 0.01 | 0.27 |  |  |  |  | 0.04 | 2.17 |
|  |  |  |  |  |  |  |  |  |
| Totals | 288 | 37 | 62 | 19 | 180 | 30 | 45 | 18 |
